# A Millimeter‐Scale Implantable Magneto–Mechano–Electric Transducer Based on BTO Piezoelectric Ceramics for Remote Wireless Electrical Stimulation of Injured Sciatic Nerves

**DOI:** 10.1002/advs.75833

**Published:** 2026-05-25

**Authors:** Yijing Wang, Jingyu Tian, Zhe Xu, Cuiling Zhang, Jingen Wu, Ming Liu, Zhilu Ye, Xiaohui Zhang

**Affiliations:** ^1^ State Key Laboratory For Manufacturing Systems Engineering Center for Mitochondrial Biology and Medicine School of Life Science and Technology International Joint Laboratory for Micro/Nano Manufacturing and Measurement Technology Key Laboratory of Biomedical Information Engineering of Ministry of Education Xi'an Key Laboratory For Biomedical Testing and High‐end Equipment Xi'an Jiaotong University Xi'an Shaanxi China; ^2^ State Key Laboratory for Manufacturing Systems Engineering Electronic Materials Research Laboratory Engineering Research Center of Spin Quantum Sensor Chips Key Laboratory of the Ministry of Education Universities of Shaanxi Province School of Electronic Science and Engineering Xi'an Jiaotong University Xi'an China

**Keywords:** battery‐free, biocompatible materials, cantilever, magneto‐mechano‐electric, no‐circuit system, sciatic nerve repair, wireless

## Abstract

Electrical stimulation is widely recognized as an effective strategy for promoting functional recovery after peripheral nerve injury. However, conventional stimulators rely on wired connections or implanted batteries, severely limiting implantability, biosafety, and clinical translation. Recent wireless, battery‐free piezoelectric systems, primarily based on ultrasound or pressure‐driven transduction, remain constrained by auxiliary equipment, working distance, and external control. Here, we report a fully wireless, circuit‐free neural stimulator built on a millimetric magneto‐mechano‐electric (MME) cantilever transducer (3 × 10 × 5 mm) with a barium titanate (BTO) piezoelectric ceramic core. Under low‐frequency alternating magnetic fields, the MME cantilever mechanically oscillates, inducing stress on the BTO element and thereby converting magnetic energy into therapeutic electrical pulses. The use of BTO minimizes potential biosafety risks. Moreover, the device operates stably at 16 cm from the magnetic source, outperforming most existing wireless stimulators. In vitro, MME‐mediated stimulation doubles PC12 neurite outgrowth and neuronal differentiation. In a rat sciatic nerve injury model, the implanted stimulator exhibits excellent biosafety and significantly enhances nerve regeneration and motor recovery, achieving a ∼1.5‐fold improvement in the sciatic functional index.

## Graphical abstract

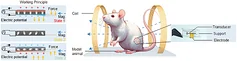

## 1 Introduction

Electrical stimulation (ES) represents a cornerstone in the fields of bioelectronic medicine and neurotherapeutics, offering non‐pharmacological treatment options for a variety of clinical conditions, including peripheral nerve injury, motor impairment, and neuropathic pain [1, 2, 3, 4, 5, 6, 7]. By delivering controlled electrical pulses, ES modulates ion channel activity on the cell membrane, initiating downstream molecular and cellular responses that influence neural function and tissue repair. Over the past decade, both preclinical and clinical studies have validated the efficacy of ES interventions in peripheral nerve regeneration and functional recovery. However, existing ES systems typically rely on external power sources connected through wired leads to implanted or surface electrodes, which constrain patient mobility, require professional oversight, and pose long‐term risks such as mechanical fatigue, material degradation, and toxic by‐product accumulation [8, 9]. Battery‐powered alternatives, while more portable, require large form factors for sustained operation and present additional challenges related to maintenance and infection.

To address these challenges, research efforts have increasingly focused on the development of wireless, battery‐free ES systems [10, 11, 12, 13, 14, 15]. Inductively coupled systems, which deliver power wirelessly from an external radio‐frequency (RF) signal source to a wearable or implantable stimulator through electromagnetic fields, have become the most prevalent approach. Although effective, these stimulators typically rely on integrated circuits or chips to stabilize the received signals and convert high transmission frequencies (>1 kHz, optimal for efficient power transfer) [16, 17] into lower frequencies suitable for neural stimulation (<300 Hz) [18, 19]. This reliance on circuitry not only complicates device architecture but also introduces reliability concerns in vivo, where exposure to conductive biofluids may lead to short‐circuiting or malfunction [20, 21, 22]. Moreover, inductive coupling generally necessitates large coils to ensure sufficient power transfer, resulting in increased device dimensions. The operational distance between the stimulator and external signal source is typically limited to a few millimeters, further restricting their practical utility [23, 24].

Piezoelectric materials, which generate electrical pulses in response to mechanical deformation, have recently emerged as a promising strategy for wireless electrical stimulation. This approach eliminates the need for integrated circuits, enabling miniature stimulator designs. Piezoelectric stimulators based on barium titanate (BTO) [25] and polyvinylidene difluoride (PVDF) [26] have shown promise in neural regeneration and the treatment of neurological disorders. However, despite their promise, current piezoelectric stimulators typically rely on self‐motion or ultrasound sources for mechanical actuation. Self‐motion often leads to inconsistent stimulation pulses and variable therapeutic results. While ultrasound provides reliable actuation, it may cause thermal damage or cavitation in human tissues, raising biosafety concerns for long‐term applications. Furthermore, ultrasound sources generally require direct contact with the skin to reduce energy loss due to impedance mismatch at the tissue–air interface. The large size of conventional ultrasound generators further limits their feasibility for home‐based use. Therefore, there remains a critical need for compact, circuit‐free wireless stimulators capable of providing safe, reliable operation over extended distances.

**FIGURE 1:**
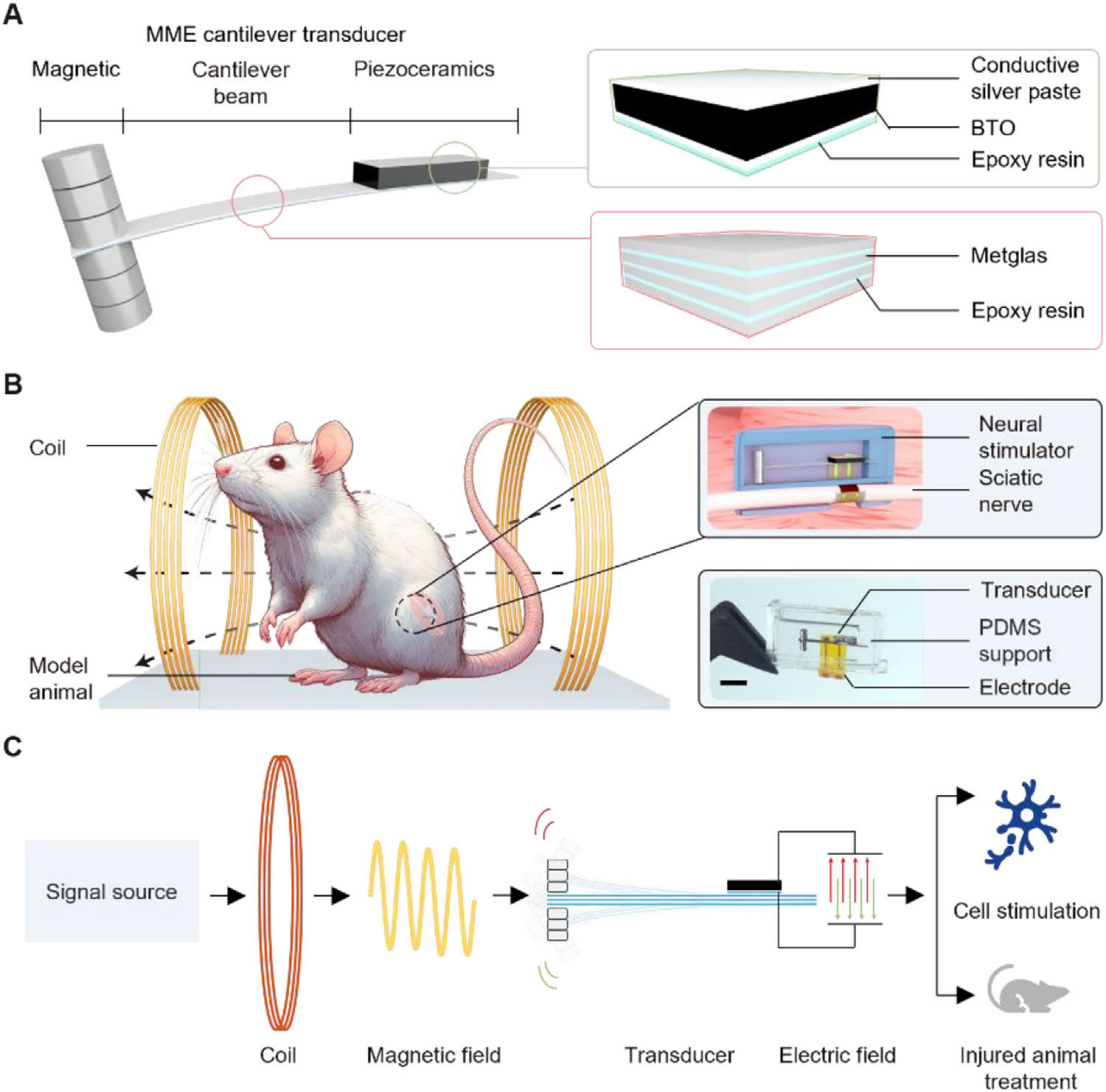
Wireless multiplexed sensing system with an ultrasoft, stretchable, wearable sensor for on‐skin perspiration monitoring. (A) Schematic illustration of the overall structure of the MME transducer, along with an exploded diagram depicting the layered composition of its principal components. (B) Photograph of the implanted device and schematic representation of a model rat implanted with the device undergoing treatment within an alternating magnetic field. (C) Workflow diagram outlining the operational process of the MME transducer system. In the photo, scale bars, 3 mm.

In this study, we developed a wireless, circuit‐free magneto‐mechano‐electric (MME) cantilever transducer constructed from highly biocompatible materials to achieve reliable neural stimulation through a compact architecture (3 × 10 × 5 mm, see Figure 1A). The MME transducer consists of a magnetically coupled mechanical cantilever that oscillates under a low‐frequency magnetic field (e.g., 60 Hz), mechanically driving a barium titanate (BTO) piezoelectric layer to directly generate voltage pulses suitable for neural stimulation. The device is encapsulated in PDMS, which also serves as a structural support, securing the cantilever within the packaging without hindering its vibration, thereby further enhancing overall biocompatibility. This MME energy conversion mechanism eliminates the need for complex circuitry, ensuring outstanding durability (signal decay <10% after 30 days of continuous operation) and excellent biocompatibility (no significant apoptosis observed after 9 days of cell culture). Compared with existing wireless stimulators constrained to millimeter‐scale operation distances, our MME stimulator achieves a remote operational range of 16 cm, substantially improving spatial flexibility. In vitro, the stimulator significantly promotes PC12 cell differentiation and neurite outgrowth, increasing the proportion of neurite‐bearing cells from 18% to 55% and extending the maximum neurite length from 22 µm to 41 µm. In a rat sciatic nerve injury model, the MME stimulator enhances axonal regeneration and motor recovery, improving the sciatic functional index from −79 to −33 and muscle mass recovery from 41% to 80%. Collectively, these results demonstrate that the MME cantilever stimulator represents a promising wireless platform for neural modulation and offers a potential solution for next‐generation implantable bioelectronic therapies (Table S1).

## 2 Results

### 2.1 Design and Characterization of the Wireless Neural Stimulator

**FIGURE 2:**
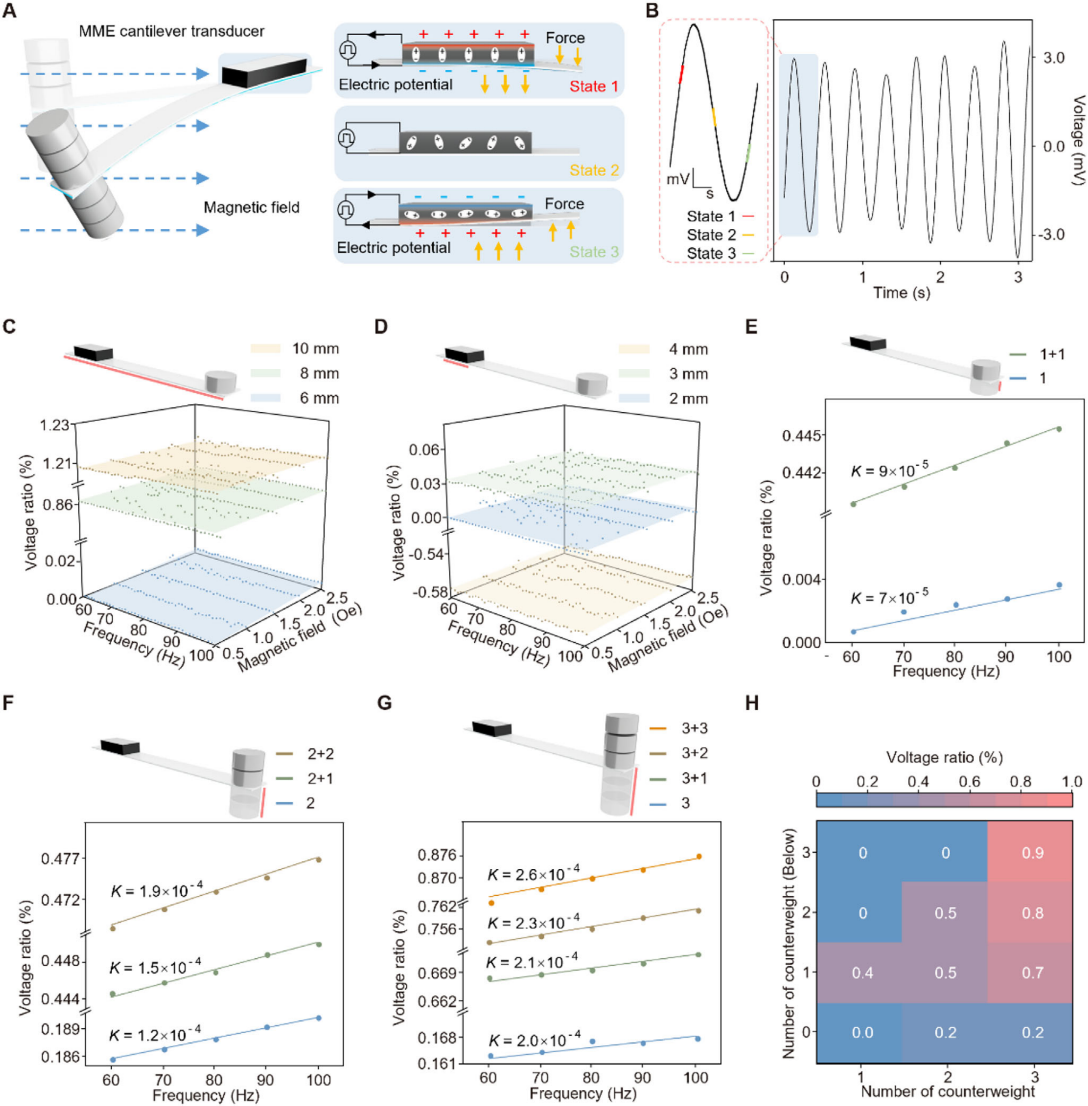
Structural optimization of the cantilever‐based MME transduction system. (A) Schematic illustration depicting the generation of electrical potential in the MME transducer under an alternating magnetic field. (B) Statistical representation of the electrical signals produced by the MME transducer when exposed to the alternating magnetic field. (C) 3D comparative plot of simulation results (surface) and experimental measurements (dots) for optimizing cantilever length (cantilever length: 6 mm, 8 mm, 10 mm; piezoelectric ceramic length: 2 mm; counterweight mode: single weight), under varying magnetic field strengths (0.5–2.5 Oe) and excitation frequencies (60–100 Hz). (D) 3D comparative plot of simulation results (surface) and experimental measurements (dots) for optimizing piezoelectric ceramic length (piezoelectric ceramic length: 2 mm, 3 mm, 4 mm; cantilever length: 10 mm; counterweight mode: single weight), under varying magnetic field strengths (0.5–2.5 Oe) and excitation frequencies (60–100 Hz). (E) Line plots comparing simulation (solid lines) and experimental (dots) results for different magnetic counterweight configurations—mode 1 (1 and 1 + 1 weights)—under fixed magnetic field (2 Oe), across frequencies of 60–100 Hz (cantilever length: 10 mm; ceramic length: 3 mm). (F) Line plots comparing simulation (solid lines) and experimental (dots) results for different magnetic counterweight configurations—mode 2 (2, 2 + 1 and 2 + 2 weights)—under fixed magnetic field (2 Oe), across frequencies of 60–100 Hz (cantilever length: 10 mm; ceramic length: 3 mm). (G) Line plots comparing simulation (solid lines) and experimental (dots) results for different magnetic counterweight configurations—mode 3 (3, 3 + 1, 3 + 2, and 3 + 3 weights)—under fixed magnetic field (2 Oe), across frequencies of 60–100 Hz (cantilever length: 10 mm; ceramic length: 3 mm). (H) Heatmap summarizing output distribution under various magnetic counterweight configurations.

The MME transducer, which serves as the core functional component of the neural stimulator, consists of three fundamental elements: (1) a magnetic proof mass or miniaturized neodymium‐iron‐boron (NdFeB) magnet; (2) a cantilever beam formed from multiple Metglas sheets bonded with epoxy resin; and (3) a piezoelectric component, i.e., BTO ceramic coated with conductive silver paste (Figure 1A; Figure S1). The MME cantilever transducer is fabricated as follows. Pre‐cut Metglas sheets are bonded with epoxy to construct the cantilever beam, while BTO ceramics are cut and silver‐coated to form electrodes. The piezoelectric ceramic is then bonded to the beam, and magnetic counterweights are affixed to complete the assembly (Figure S2). The implantable wireless neural stimulator is constructed by integrating the MME transducer unit, which converts low‐frequency magnetic fields into therapeutic electrical signals, with a flexible electrode for delivering stimulation to the injured sciatic nerve, and a custom‐fabricated polydimethylsiloxane (PDMS) scaffold that securely positions the stimulator and the damaged nerve and provides adequate space for cantilever oscillation (Figure 1B, right). For therapeutic stimulation, rats implanted with the neural stimulator are positioned within a spatially uniform alternating magnetic field generated by a 1D Helmholtz coil system (Figure 1B, left). To further evaluate the stability of the excitation environment, we perform numerical simulations and experimental measurements of the magnetic field distribution within the effective working region of the stimulation setup. The results confirm that the magnetic field remains sufficiently uniform in this region and that the implanted device maintains stable output under representative spatial variation (Figure S3). The alternating magnetic field induces mechanical oscillation of the magnetic proof mass, exciting bending vibrations of the cantilever. Through cyclic deformation of the cantilever, involving elongation (state 1), recovery (state 2), and compression (state 3), mechanical stress is applied to the piezoelectric ceramic, thereby inducing an electric potential difference via the direct piezoelectric effect (Figures 1C and 2A). The resulting potential difference drives charge migration between the high‐ and low‐potential regions, generating a periodic electrical output (Figure 2,A,B). This MME energy conversion mechanism produces electrical output at the same frequency as the external magnetic field (tens to hundreds of hertz), matching the optimal range for electrical stimulation to promote nerve repair. The generated signals can thus be directly delivered to the injury site through conductive leads and electrodes. Furthermore, unlike conventional inductive or ultrasound‐based coupling methods, which require the transmitter to remain near or in contact with the skin (typically within a few centimeters), this approach enables an extended operation range of 16 cm, allowing treated animals to move freely within a defined space.

The performance of the MME cantilever transducer is systematically investigated under varying dimensional and structural configurations. Both experimental measurements and COMSOL Multiphysics simulations are conducted to ensure robust and validated results. Initially, we examine the effect of cantilever length on output voltage, keeping the cantilever width fixed at 1 mm, the BTO ceramic dimensions at 1 × 2 × 1 mm, and employing a single‐unit magnetic proof mass. Cantilevers with lengths of 6, 8, and 10 mm are tested under magnetic field intensities ranging from 0.5 to 2.5 Oe and frequencies between 60 and 100 Hz. The peak output voltages for the 6 mm, 8 mm, and 10 mm cantilevers reach 3.9 mV, 4.2 mV, and 4.5 mV, respectively, under a 2.5 Oe, 100 Hz magnetic field. Experimental data (dots) show excellent agreement with simulation results (surfaces) (Figure 2C), demonstrating that longer cantilevers consistently produce higher output voltages. In addition, the output voltage exhibits a moderate increase with rising magnetic field intensity and frequency. Similar trends are observed when varying frequencies under a constant magnetic field strength (e.g., 2.0 Oe) or altering magnetic field strength at a fixed frequency (e.g., 60 Hz); in both cases, the output increases with cantilever length. The 10 mm cantilever group consistently exhibits the highest output voltage, and the observed responses follow a functional relationship with the corresponding parameters (Figure S4A,B). These consistent results across different magnetic parameters highlight the system's stable and repeatable response characteristics, thereby confirming the reliability of the device and enabling further optimization of the mechanical structure.

In subsequent investigations, we systematically examine the influence of BTO ceramic length on the output performance under controlled conditions, wherein the piezoelectric ceramic width is fixed at 1 mm, the cantilever dimensions are maintained at 1 × 10 mm, and a single permanent magnet is loaded. As shown in the result (Figure 2D), the output voltage of the MME increases with the length of the BTO element, reaching a peak at a length of 3 mm. Under the condition of 100 Hz and 2.5 Oe, the output voltages corresponding to BTO lengths of 2 mm, 3 mm, and 4 mm are approximately 4.5 mV, 4.7 mV, and 4.3 mV, respectively. A similar trend is observed under fixed magnetic field strength (e.g., 2.0 Oe) and frequency (e.g., 60 Hz), further confirming the critical role of BTO length in modulating the MME output performance (Figure S5A,B). Notably, the device integrated with a 3 mm‐long BTO ceramic consistently demonstrates the highest output voltage across all testing conditions. This phenomenon is likely attributed to the balance between the piezoelectric response area and mechanical vibrational efficiency. Within the range of 2–3 mm, the increase in ceramic length enhances the effective area capable of responding to mechanical oscillations, thereby boosting output voltage. However, when the ceramic length exceeds 3 mm, the excessive extension appears to compromise the cantilever's effective vibrational amplitude, which in turn diminishes the electromechanical conversion efficiency of the piezoelectric element.

In addition to the dimensions of the cantilever and piezoelectric material, the configuration of the NdFeB magnets plays an important role in determining the output voltage. To evaluate this effect, we vary the number of magnets positioned above and below the cantilever, while maintaining fixed cantilever and piezoelectric dimensions (1 × 10 mm Metglas, 1 × 3 × 1 mm BTO). Three magnetic loading configurations are examined: one magnet above (with zero or one magnet below; 1 + 0 or 1 + 1), two magnets above (with zero, one, or two magnets below; 2 + 0, 2 + 1, or 2 + 2), and three magnets above (with zero to three magnets below; 3 + 0, 3 + 1, 3 + 2, or 3 + 3). The experimental data closely align with the simulation results (Figure 2E,G), confirming that across different magnetic configurations, increasing the total number of magnets substantially enhances both the output voltage and the voltage–frequency response sensitivity (Figures S6A, S7A, and S8A). And a similar trend is observed under fixed magnetic field strength (e.g., 2.0 Oe) and frequency (e.g., 60 Hz), further corroborating that the magnetic counterweight, within a certain range, can effectively modulate the output performance of the MME (Figures S6B and S8B). Specifically, under the boundary condition, a 2.5 Oe, 100 Hz magnetic field, the 3 + 3 magnet configuration yields the highest output voltage (6.1 mV), outperforming other configurations such as 1 + 1 (5.1 mV) and 2 + 2 (5.2 mV). Taken together, the data clearly demonstrate that the MME device configuration incorporating a 1 × 10 mm Metglas cantilever, a 1 × 3 × 1 mm BTO piezoelectric element, and a 3+3 magnetic array yields the highest voltage output among all tested architectures (Figure 2H). This configuration not only maximizes energy conversion efficiency but also ensures superior system stability, thereby offering valuable guidance for the rational design of next‐generation magnetoelectric devices. This design achieves a maximum voltage output of at least 6 mV (Based on the final design of the therapeutic electrode, the electric field intensity can be precisely regulated to ∼60 mV/cm.) within the frequency range of 60–100 Hz, and under a reasonable electrode configuration, the resulting electric field strength falls well within the effective threshold range required for neural stimulation (50–100 mV/cm) [27, 28].

Based on these data, it is evident that the output of the transducer continues to increase with either a greater number of magnetic counterweights or a further extension of the cantilever length. However, practical constraints associated with in‐vivo implantation necessitate a trade‐off between device volume and electrical output, highlighting the importance of structural optimization for clinically relevant applications. Therefore, the practical efficacy of this configuration in bioelectronic stimulation applications requires further validation through in vitro stimulation experiments described in the subsequent section.

### 2.2 Optimization of Electrical Stimulation Parameters

**FIGURE 3:**
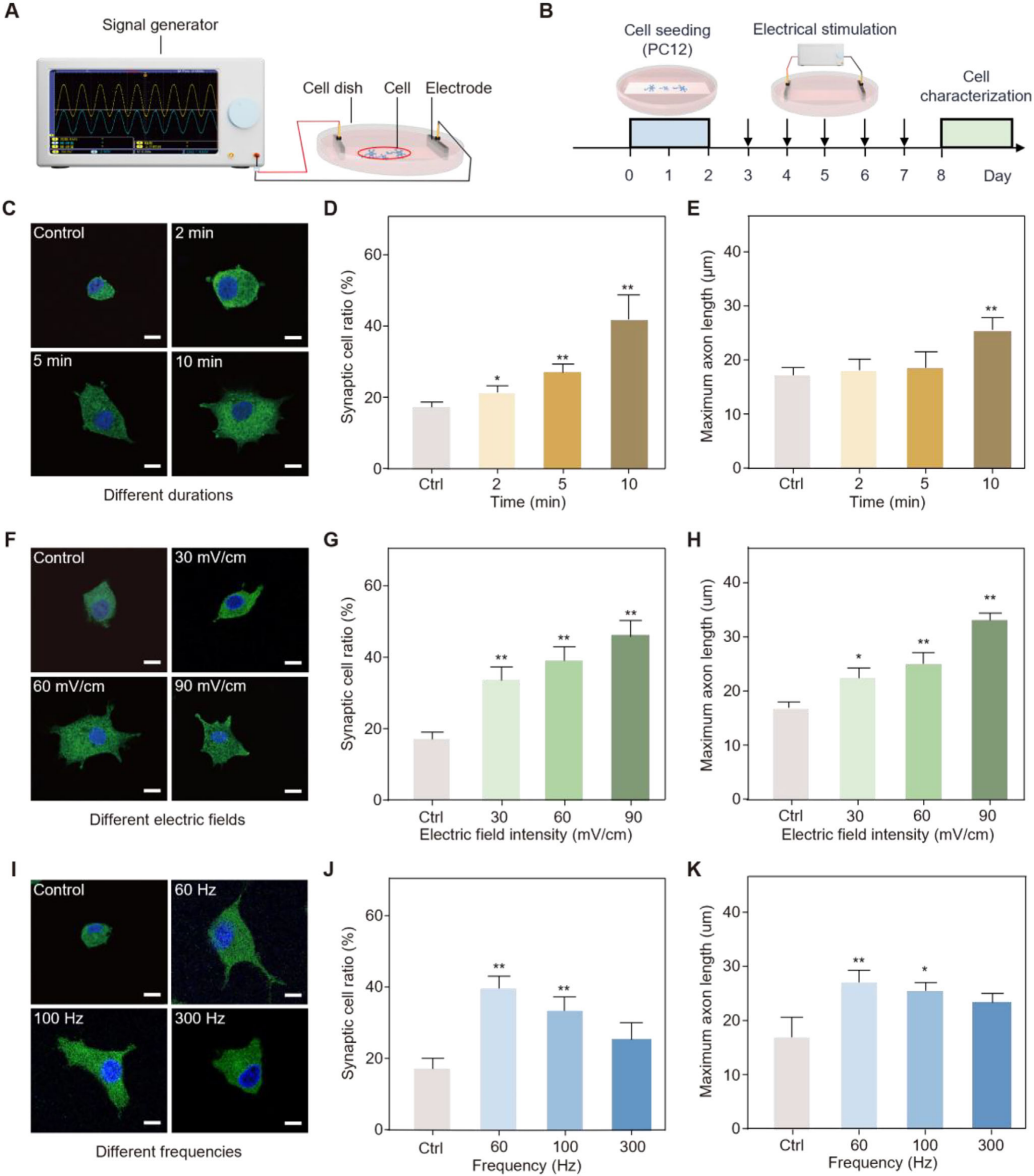
Optimization of electrical stimulation parameters. (A) Schematic illustration of the experimental setup for electrical stimulation of PC12 cells using a signal generator. The two electrodes are separated by 1 cm. Under this configuration, signal generator outputs of 30 mV, 60 mV, and 90 mV generate electric field strengths of 30, 60, and 90 mV/cm, respectively. (B) Design overview for optimization of stimulation parameters, including stimulation duration (2, 5, and 10 min per session), electric field strength (30, 60, and 90 mV/cm), and field frequency (60 Hz, 100 Hz, and 300 Hz). (C) Representative immunofluorescence images of PC12 cells subjected to varying stimulation durations, including 2, 5, 10 min per session (field strength: 60 mV/cm; field frequency: 60 Hz). (D) Quantification of the proportion of neurite‐bearing cells under different stimulation durations. (E) Statistical analysis of maximal neurite length of PC12 cells across stimulation durations. (F) Representative immunofluorescence images of PC12 cells under varying electric field strengths, including 30 mV/cm, 60 mV/cm, 90 mV/cm (stimulation duration: 10 min per session; field frequency: 60 Hz). (G) Quantification of neurite‐bearing cell proportion under different field strengths. (H) Statistical analysis of maximal neurite length under different field strengths. (I) Representative immunofluorescence images of PC12 cells under different field frequencies, including 60 Hz, 100 Hz, and 300 Hz (stimulation duration: 10 min per session; field strength: 60 mV/cm). (J) Quantification of neurite‐bearing cell proportion under various field frequencies. (K) Statistical comparison of maximal neurite length across different field frequencies. In all immunofluorescence images: blue, Hoechst, nuclear stain; green, βIII‐Tubulin, neuronal marker. Scale bars, 10 µm. Statistical significance was determined by comparison between the stimulation and control groups: *p* ≤ 0.05 (^*^), *p* ≤ 0.01 (^**^).

Enhancement of neuronal activity at the cellular level is essential for promoting peripheral nerve regeneration. Therefore, prior to conducting in vivo studies using nerve injury models, we perform in vitro electrical stimulation experiments to determine the optimal stimulation parameters for facilitating neurite outgrowth. Pheochromocytoma PC12 cells serve as the research subjects in this study because they are classic engineered cells that play a crucial role in academic research, such as in neural signal transmission and neural regeneration [29, 30]. The evaluated parameters include stimulation duration (2, 5, and 10 min per session), electric field strength, and stimulation frequency (60 Hz, 100 Hz, and 300 Hz). Based on the maximum output voltage of the MME transducer (at least 6 mV) and considering an electrode spacing of approximately 1 mm in subsequent animal models, the system is capable of generating an electric field of 60 mV/cm. Accordingly, the experimental electric field strengths are set at 30 mV/cm, 60 mV/cm, and 90 mV/cm by controlling the electrode spacing at 10 mm during stimulation (Figure 3B). To ensure precise control of stimulation conditions, electrical signals are delivered using a signal generator (AFG1062) in a custom stimulation chamber (Figure 3A; Figure S16A for link block diagram). Cells in the stimulation groups receive daily electrical stimulation for five consecutive days (Figure 3B), while the control groups are cultured in standard dishes without stimulation.

The effect of stimulation duration is first evaluated, with electric field intensity fixed at 60 mV/cm and frequency at 60 Hz. βIII‐Tubulin is used here as a commonly adopted neuronal cytoskeletal marker for evaluating neurite outgrowth and neuronal‐like differentiation in PC12 cells [31, 32, 33]. (Figure 3C; nuclei stain blue with Hoechst, neurites labeled green with βIII‐Tubulin). Quantitative analysis reveals that all three stimulation durations enhance the neurite‐bearing cell ratio and maximal neurite length in PC12 cells compared to the control. Particularly, longer stimulation durations lead to more significant enhancement, with 10‐min daily stimulation achieving the highest values (40% neurite‐bearing cell ratio and 27 µm maximal neurite length), without inducing morphological abnormalities or other observable adverse effects (Figure 3D,E). Therefore, 10 min of stimulation is used for subsequent experiments.

We next explore the effect of electric field intensity on neural stimulation outcomes. Electric fields of 30 mV/cm, 60 mV/cm, and 90 mV/cm are applied, while maintaining a fixed frequency of 60 Hz and a stimulation duration of 10 min (Figure 3F). Similarly, immunofluorescence staining and quantitative analysis show a consistent increase in neurite‐bearing cell ratio and maximal neurite length under all field intensities, compared to the unstimulated control. Higher electric field intensities result in more significant enhancement. Specifically, electrical stimulation at 90 mV/cm results in the highest proportion of neurite‐bearing cells (45%) and the longest neurite outgrowth (35 µm) (Figure 3G,H). However, in practical applications, two critical limitations must be addressed. First, the miniaturization requirements of the MME device inherently constrain the output voltage to approximately 6 mV, which is lower than the optimal stimulation intensity. Second, the use of a 1D coil for magnetic actuation imposes a significant risk: prolonged exposure (>10 min) to strong magnetic fields (>2.0 Oe) can lead to excessive mechanical loading, potentially causing irreversible systemic damage to the device. Considering these findings, the development of a compact external magnetic stimulation system with both high‐field output and substantial load‐bearing capacity emerges as a critical engineering challenge that must be overcome to facilitate the clinical translation of this technology. Therefore, considering both the efficacy of stimulation and the physical constraints of device miniaturization, 60 mV/cm is identified as the optimal stimulation condition for further experimentation. This setting maintains overall system safety while still achieving notable biological outcomes, specifically a 40% neurite‐bearing cell ratio and neurite extension reaching 26 µm.

We further evaluate the effect of stimulation frequency while maintaining a constant stimulation duration (10 min per session) and electric field intensity (60 mV/cm). Stimulation frequencies of 60 Hz, 100 Hz, and 300 Hz are applied (Figure 3I). Immunofluorescence imaging reveals enhanced neurite outgrowth and extension under all frequencies compared to controls. However, the response exhibits a biphasic profile. Specifically, as the stimulation frequency increases from 60 Hz to 300 Hz, the maximal neurite length gradually decreases (Figure 3J). A similar trend is observed in the neurite‐bearing cell ratio (Figure 3K). These findings indicate that excessively high stimulation frequencies (e.g., hundreds of hertz or above) provide limited benefit to neural cells, consistent with previous reports [34]. Based on these results, 60 Hz is identified as the optimal stimulation frequency. Considering the stimulation outcomes, physical constraints, and operational feasibility, the final MME cantilever transducer design and stimulation protocol are established. The optimized configuration includes a transducer width of 1 mm, a cantilever length of 10 mm, a piezoelectric ceramic length of 3 mm, and a 3 + 3 magnetic loading arrangement. The stimulation parameters are set at 60 mV/cm, 60 Hz, for 10 min per session. These specifications provide a balance between device miniaturization and stimulation performance and are employed subsequently in vitro and in vivo studies.

### 2.3 In Vitro Validation of the Therapeutic Efficacy of the MME Cantilever Transducer

**FIGURE 4:**
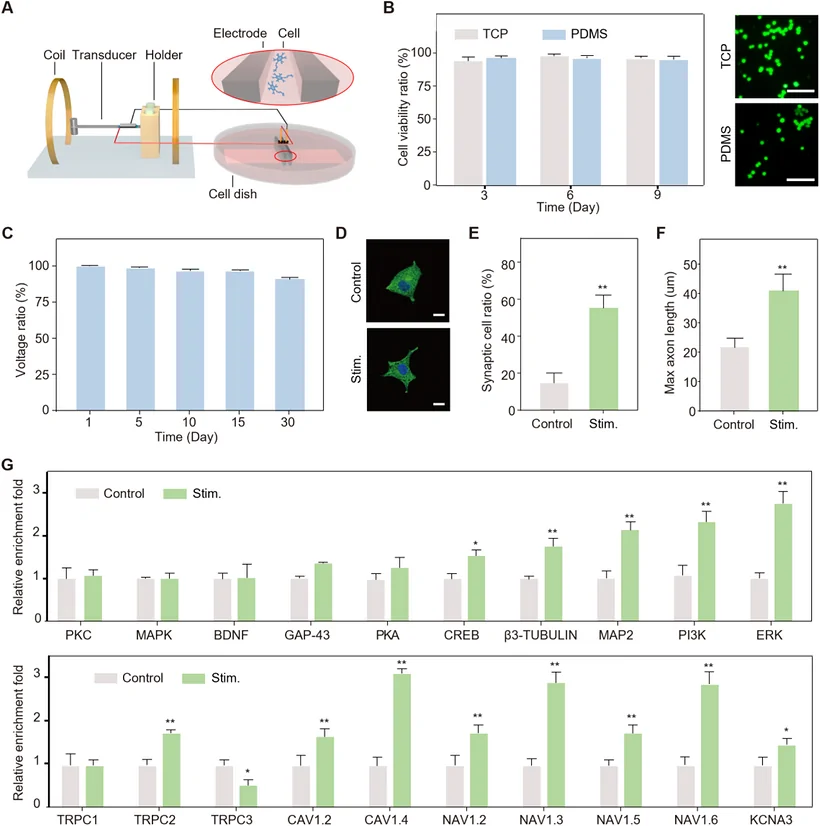
In vitro cellular response to stimulation from the cantilever‐based MME transducer. (A) Schematic diagram of the experimental setup for in vitro cell stimulation using the cantilever‐based MME device. The electrodes are separated by 1 mm, and a device output of 6 mV generates an electric field of 60 mV/cm. (B) Biocompatibility assessment of the MME device substrate, including quantitative analysis and live/dead cell staining images (green: Calcein‐AM, live cell marker; red: propidium iodide (PI), dead cell marker). (C) Quantitative analysis of the long‐term output stability of the MME device over 30 days. (D) Immunofluorescence staining of PC12 cells after continuous stimulation with the MME device (Stim.) for 5 days (blue: Hoechst; green: βIII‐Tubulin). (E) Quantification of the proportion of neurite‐bearing PC12 cells after 5 days of continuous stimulation. (F) Statistical analysis of the maximum neurite outgrowth length in PC12 cells following 5‐day stimulation. (G) Relative mRNA expression levels of PC12 cells after 5 days of stimulation. Scale bars, 10 µm. Statistical significance was determined by comparison between the stimulation and control groups: *p* ≤ 0.05 (^*^), *p* ≤ 0.01 (^**^).

In this section, the efficacy of our MME cantilever‐based neural stimulator in promoting PC12 differentiation and neurite outgrowth in vitro is evaluated. The MME cantilever transducer is securely mounted on a 3D‐printed holder and positioned in the magnetic field generated by the Helmholtz coil, with its output connected via electrodes to a slide seeded with PC12 cells (Figure 4A; Figure S16B for link block diagram). Prior to stimulation, we evaluate the biocompatibility and long‐term stability of the PDMS‐encapsulated transducer; this same device configuration is used in subsequent in vivo studies. To assess biocompatibility, PC12 cells are cultured on the encapsulating material (PDMS group) and on standard culture dishes (TCP group) for nine consecutive days. Live/dead staining conducted on days 3, 6, and 9 reveals no significant difference in cell viability or morphology between the two groups (Figure 4B). Additionally, the stimulator demonstrates stable voltage output throughout a 30‐day continuous operation period (Figure 4C), verifying its operational stability over the timeframe intended for in‐vitro stimulation. We further characterize the terminal response of the implantable MME device by measuring voltage outputs under different load conditions (e.g., air, saline, muscle, and neural tissue). The device maintains stable periodic voltage waveforms under these conditions (Figure S9). To verify electrical delivery at the target stimulation site, we measure the current delivery to neural tissue (Figure S10). The measured alternating current waveform shows a peak‐to‐peak current of 73.1 µA. Numerical integration gives positive and negative phase charges of 14.5 nC and −13.8 nC, respectively, corresponding to a current density of 11.6 mA/cm^2^. These results confirm measurable current delivery and balanced biphasic charge transfer to the target neural tissue, consistent with previous piezoelectric and magnetoelectric bioelectronic studies [35, 36, 37, 38]. Subsequently, electrical stimulation is applied to PC12 cells under optimized parameters (60 Hz, 60 mV/cm, 10 min per day for five consecutive days; Figure S15A for workflow). Immunofluorescence analysis reveals a significant increase in neurite outgrowth in the stimulation group compared to controls (Figure 4D). Quantitative assessment further demonstrates a marked rise in both the percentage of neurite‐bearing cells (Figure 4E; control, 18%; stimulation, 55%) and maximal neurite length (Figure 4F; control, 22 µm; stimulation, 41 µm), underscoring the efficacy of the MME cantilever‐based stimulator in promoting neuronal differentiation and outgrowth.

To further clarify the molecular basis of the observed neuronal enhancement, we perform quantitative gene expression analysis of markers related to neuronal structure, function, and intracellular signaling (Figure 4G). As anticipated from the immunofluorescence results, we observe significant upregulation of βIII‐Tubulin and MAP2 transcripts (1.7‐fold and 2.1‐fold, respectively), two cytoskeletal proteins essential for microtubule dynamics and neurite outgrowth. This transcriptional activation aligns closely with the morphological evidence of neurogenesis enhancement [33, 39, 40, 41]. In parallel, we examine the expression levels of key downstream effectors involved in neuronal proliferation and survival. The transcription factor CREB exhibits a 1.56‐fold increase, accompanied by increased expression of signaling intermediates PI3K (2.3‐fold) and ERK (2.8‐fold), indicating their established roles in promoting neural cell growth and synaptic plasticity [42, 43, 44, 45, 46].

In the aspect of electrophysiological functionality, the upregulation of voltage‐gated sodium channels NaV1.2 (1.7‐fold) and NaV1.3 (2.9‐fold) suggest enhanced neuronal excitability [47, 48]. Concurrently, increased expression of NaV1.5 (1.7‐fold) and NaV1.6 (2.9‐fold) may reflect advanced cellular maturation and functional preparedness [49, 50, 51]. Regarding potassium channels, a modest increase in KCN3 expression (1.5‐fold) may indicate improved ion homeostasis and neuroprotection, given its currently reported association with neurophysiological integrity [52]. We also observe upregulation of TRPC2 (1.7‐fold), a calcium‐permeable channel responsible for modulating sensory signaling and G‐protein‐coupled receptor (GPCR) interactions [53, 54], suggesting enhanced maintenance of neuronal functionality. Furthermore, CaV1.2 and CaV1.4, both key regulators of synaptic signal transduction, are significantly upregulated (1.7‐fold and 3.1‐fold, respectively), which may suggest the role of calcium signaling in promoting neurite complexity and synaptic activity [55, 56]. In contrast, TRPC3 exhibits a downregulated expression (0.5‐fold), consistent with previous studies linking its suppression to reduced oxidative stress and improved cell viability [57, 58], thereby further supporting the protective role of electrical stimulation in our experiments. Collectively, the cantilever‐based stimulation platform developed in this study not only significantly enhances neurite outgrowth and extension but also activates gene networks involved in neuronal development, functional maturation, and cellular homeostasis. These findings highlight the promising therapeutic potential of bioelectrically driven stimulation platforms in the context of neural tissue engineering and regenerative medicine.

### 2.4 In Vivo Validation of the Wireless Neural Stimulator in a Rat Model

**FIGURE 5:**
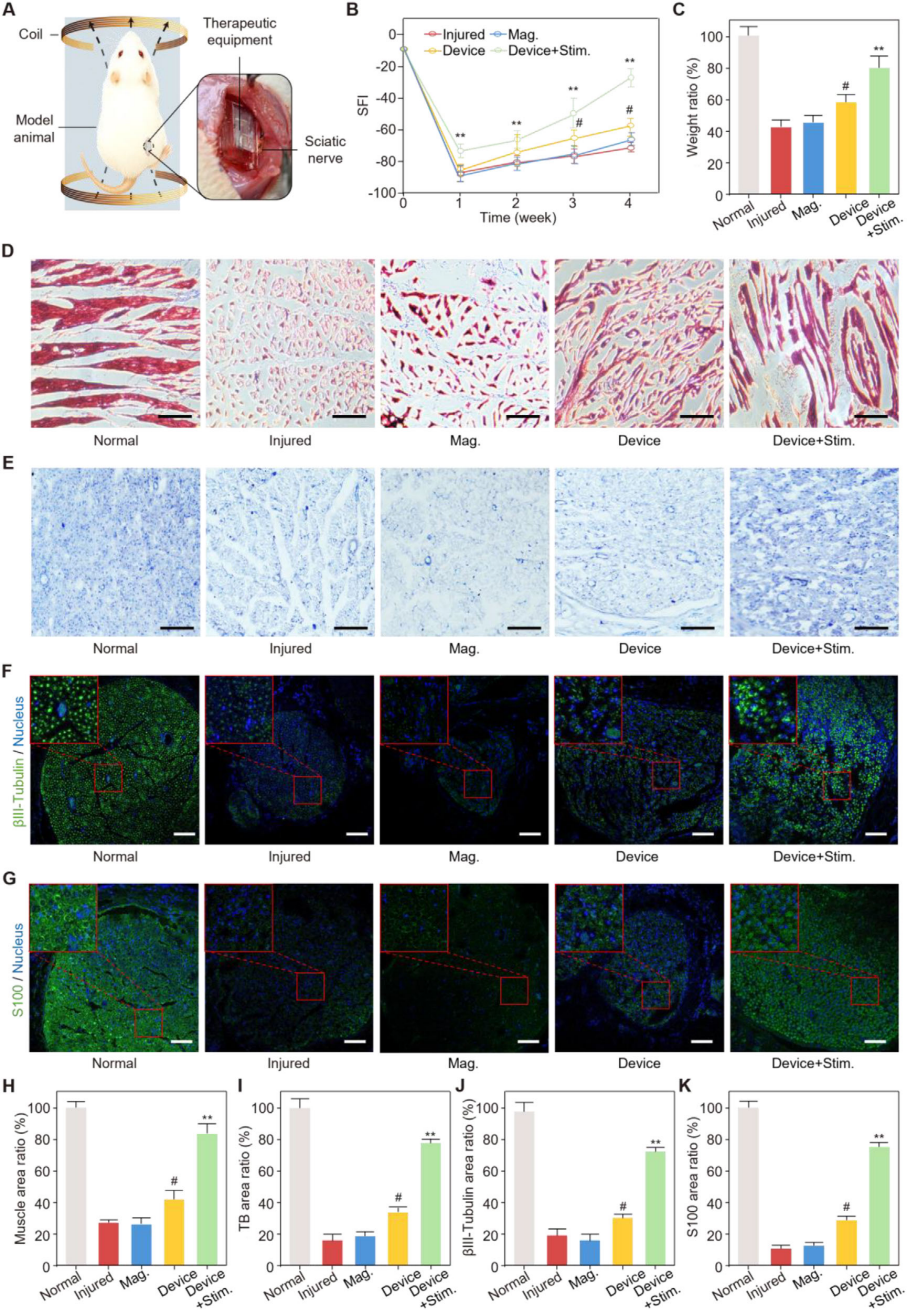
Restoration of sciatic nerve injury in rat models using wireless electrical stimulation devices. (A) Schematic illustration and corresponding photograph of the implanted device in the animal model. (B) Quantitative analysis of the sciatic functional index (SFI) in the injury group (Injured), magnetic‐field‐only group (Mag.), device‐stimulation group (Device+Stim.), and device‐only group (Device) over 1‐, 2‐, 3‐, and 4‐weeks post‐injury. (C) Comparison of gastrocnemius muscle mass among the injury (Injured), magnetic‐field‐only (Mag.), device‐stimulation (Device+Stim.), and device‐only (Device) groups at week 4 post‐repair. (D) Representative Masson's trichrome staining images showing the cross‐sectional morphology of gastrocnemius muscle in each group. (E) Representative Toluidine Blue staining images of sciatic nerve cross‐sections in each group. (F) Representative immunofluorescence staining of S100 protein in sciatic nerve tissue in each group. (blue: Hoechst; green: S100, and the image with a 2.5x magnification is in the upper left corner). (G) Representative immunofluorescence staining of βIII‐Tubulin in sciatic nerve sections in each group. (blue: Hoechst; green: βIII‐Tubulin, and the image with a 2.5x magnification is in the upper left corner). (H) Quantification of the muscle fiber area ratio based on Masson staining analysis in each group. (I) Quantification of stained nerve fiber area from Toluidine Blue‐stained sections in each group. (J) Quantification of S100‐positive area relative to total nuclear area in each group. (K) Quantification of βIII‐Tubulin‐positive staining area in each group. Scale bars, 100 µm. Statistical comparisons were made between the device‐stimulation and the injury group (^*^), and between the device‐only group and the injury group (^#^). *p* ≤ 0.01 (^**^), *p* ≤ 0.05 (^#^).

To demonstrate the therapeutic efficacy in vivo, the MME cantilever transducer is encapsulated with a custom‐designed PDMS holder (Figure 4B, and the biological compatibility is verified in an in vitro cell experiment. A nerve conduct to precisely position the nerve and electrodes, ensuring reliable stimulation delivery (Figure S10, the flowchart of the device's preparation process). The established neural simulator is in compact dimensions for implementation (3 × 10 × 5 mm). A rat model of sciatic nerve crush injury is employed, where the sciatic nerve is exposed under anesthesia and compressed to induce mechanical injury. For stimulator implantation, the compressed sciatic nerve is passed through the conduct of the implantable stimulator. The stimulation electrodes are positioned so that both ends of the injury site encounter the embedded electrodes. The surgical wound is subsequently closed in layers. The rats are assigned to four groups: a normal group (no nerve injury, short for Normal), an injured group (sciatic nerve crush injury with no stimulator or electrical stimulation, short for Injured), an magnetic‐field‐only group (sciatic nerve crush injury exposed to the external magnetic field but without implantation of the MME device, short for Mag.), a device‐only group (sciatic nerve crush injury with stimulator implantation but no electrical stimulation, short for Device), and a device‐stimulation group (sciatic nerve crush injury with stimulator implantation and electrical stimulation, short for Device+Stim.; *n* = 3 for each group). All rats are housed under identical conditions to ensure a fair comparison. The device‐stimulation group receives electrical stimulation provided by our wireless neural stimulator under an external magnetic field of 60 Hz, 2 Oe (Figure 5A; Figure S16B for link block diagram). The stimulation is applied 10 min per session, once every two days for 4 weeks (Figure S15B for schematic illustration of the experimental workflow). These stimulation conditions are consistent with previous optimized parameters. The 4‐week postoperative observation period is selected according to previous studies and recent methodological reviews of rat sciatic nerve crush injury [59, 60, 61], which indicate that this time window is sufficient for distinguishing recovery differences among experimental groups and for reliable evaluation of functional and histological outcomes.

As sciatic nerve injury directly impairs muscle and motor function, functional recovery is assessed using the sciatic functional index (SFI), a widely recognized metric for evaluating hindlimb motor performance in rodents. After 4 weeks of recovery, the device‐stimulation group exhibits significantly higher SFI values compared to both the injured and device‐only groups, indicating improved motor recovery (Figure 5B; Figure S11 for footprint images). These findings are further supported by representative hindlimb images (Figure S12). Consistent with these functional outcomes, the gastrocnemius muscle mass in the device‐stimulation group reaches 82% of that in normal controls, suggesting effective prevention of denervation‐induced muscle atrophy. By contrast, the injured, magnetic‐field‐only group and device‐only groups retain only 41%, 43%, and 59% of normal muscle weight, respectively, reflecting persistent functional impairment (Figure 5C). Histological analysis using Masson's trichrome staining further supports these results (Figure 5D), revealing that the muscle fiber area is preserved at 82% in the device‐stimulation group, whereas the injured, magnetic‐field‐only, and device‐only groups exhibit reduced fiber area (28%, 27%, and 41%, respectively), indicating inadequate recovery in the absence of stimulation (Figure 5H). The corresponding representative gross images of the harvested gastrocnemius muscle tissues from each group are provided in Figure S13. We note that the device‐only group also exhibits a slight functional improvement, which may be attributed to the presence of the conduit structure within the device. Such structures have been shown in numerous studies to facilitate nerve regeneration to a certain extent [62, 63].

To investigate the neural mechanisms underlying functional improvement, we examine the sciatic nerve segment proximal to the gastrocnemius muscle. Toluidine blue staining reveals significant enhancement in the myelinated nerve fiber area in the device‐stimulation group (76%), compared to those in the injured (17%), magnetic‐field‐only (18%), and device‐only (37%) groups (Figure 5E,I). This morphological trend is further supported by immunofluorescent staining of βIII‐Tubulin, a marker of neuronal integrity and regeneration. The βIII‐Tubulin expression ratio of 75% is observed in the device‐stimulation group, while those in the injured, magnetic‐field‐only, and device‐only groups are merely 20%, 17%, and 31%, respectively, confirming enhanced neuronal regeneration following stimulation (Figure 5F,J). Furthermore, we evaluate the nerve repair process by examining glial cell responses, a key indicator of regeneration. Immunofluorescence staining for S100, a glial‐specific marker, demonstrates a significantly higher S100‐positive ratio in the device‐stimulated group (74%) than in the injury (11%), magnetic‐field‐only (13%), and device‐only (26%) groups (Figure 5G,K). The corresponding enlarged images of the immunofluorescence‐stained tissue sections are placed in the upper‐left corner of the original images, and all the enlarged images of the stained neural tissue sections are provided in the Supplementary Materials (Figure S14). These findings indicate that the MME cantilever‐based stimulator, with applied stimulation, modulates glial activity to facilitate nerve repair, consistent with previous reports [64, 65]. As a conclusion, our wireless MME electrical stimulation platform facilitates peripheral nerve repair by simultaneously modulating key protein activity and associated gene expression in both neurons and glial cells at the injury site. We note that these therapeutic effects arise primarily from device‐mediated electrical stimulation, rather than from the external magnetic field alone or device implantation alone. This coordinated regulation significantly accelerates functional recovery and motor performance in the rat injury model within a relatively short period. Therefore, our MME stimulation system, which integrates a mechanical‐to‐electrical energy conversion mechanism with a minimally invasive implantation design, offers a promising technological strategy for neuromodulation‐based therapeutic interventions.

## 3 Discussion

In this study, we have developed a wireless, millimeter‐scale MME cantilever‐based neural stimulator capable of delivering reliable electrical stimulation without reliance on complex electronic circuitry. This device employs a magnet‐loaded cantilever that transduces low‐frequency magnetic stimuli into mechanical vibration, which are subsequently converted into electrical outputs via a piezoelectric BTO element. Additional circuitry could help alleviate the source‐load mismatch between a high‐impedance piezoelectric transducer and low‐impedance biological tissue, as reported in Chen's work [66]. However, the objective of the present work is to develop a millimeter‐scale, circuit‐free stimulator with a simple architecture and minimal rigid components. Therefore, after considering the trade‐off between output conditioning and device miniaturization, no post‐transducer circuit is included in the present design. Circuit‐free architecture enables robust operation in implanted environments and achieves extended wireless transmission distances (tens of centimeters), beyond the limitations of conventional near‐field inductive systems. We have optimized both the device architecture and stimulation parameters to ensure effective therapeutic performance. We have demonstrated that the stimulator significantly promotes neurite extension in PC12 cells and increases the proportion of neurite‐bearing cells. In a sciatic nerve injury model, the stimulator has shown effectiveness in promoting nerve regeneration (the myelinated nerve fiber area increased from 17% in the untreated group to 76% following stimulation), mitigating muscle atrophy (the muscle mass increased from 41% in the untreated group to 80% following stimulation), and restoring motor function (SFI improved from −79 in the untreated group to −33 following stimulation). This circuit‐free, wireless stimulation platform offers a promising strategy for bioelectronic therapy and establishes a foundation for next‐generation, wireless, and non‐invasive biomedical systems.

## 4 Materials and Methods

### 4.1 Fabrication of the Cantilever‐Based MME Transducer

The magneto‐mechano‐electric (MME) transducers were fabricated using a multilayer cantilever architecture, comprising a piezoelectric ceramic core (BTO), an amorphous metal substrate (Metglas), and a magnetic proof mass (NdFeB). Initially, Metglas ribbons were cut into strips with dimensions of 1 × 6 mm, 1 × 8 mm, and 1 × 10 mm, which were subsequently bonded together using a two‐part epoxy resin (resin: hardener  =  10:1) to form the elastic backbone of the cantilever. The assembled structures were clamped and cured at room temperature to ensure structural stability. BTO ceramics were precisely machined into block elements with dimensions of 1 × 2 × 1 mm, 1 × 3 × 1 mm, and 1 × 4 × 1 mm using a precision cutter. Conductive silver paste was applied to the surfaces orthogonal to the polarization axis to form electrode contacts, and 0.1 mm enameled copper wires were affixed to each electrode surface as electrical leads. The assembled ceramic electrodes were cured at room temperature. The prepared BTO ceramics with defined electrodes were then bonded onto the pre‐cured Metglas substrates using epoxy resin and cured again at room temperature for approximately 24 h. A NdFeB magnet was attached to the free end of the cantilever to serve as the magnetic proof mass. The resulting MME cantilever transducers were employed for subsequent structural optimization and in vitro parameter tuning experiments. Devices demonstrating optimal output performance were selected for integration into implantable wireless stimulation systems for further use in animal studies.

### 4.2 Fabrication of the Wireless MME Stimulation Device

The PDMS fixation structure was fabricated using a custom‐designed mold. A longitudinal slit was created along the central axis of the bottom conduit to facilitate the placement of the sciatic nerve. The optimized MME transducer was anchored at the designated fixed end of the PDMS fixture. Subsequently, PDMS sidewalls pre‐embedded with flexible electrodes were aligned and bonded to the primary fixation unit. After complete curing of the PDMS interfaces, conductive silver paste was applied to bond the output leads of the MME transducer to the corresponding terminals of the FPC electrodes. The remaining open surfaces of the fixation structure were then sealed using thin PDMS sheets to complete encapsulation and prevent fluid ingress during in vivo operation. The detailed fabrication procedure is illustrated in the equipment construction flowchart (Figure S10). Prior to surgical implantation, all devices were sterilized by wiping with 75% ethanol followed by 20 min of ultraviolet irradiation to ensure aseptic conditions.

### 4.3 Simulation of the Cantilever‐Based MME Transducer

The performance of the cantilever‐based MME transducer was analyzed through numerical simulations using COMSOL Multiphysics. The simulation incorporated solid mechanics, magnetic fields, and electrostatic fields, and a cubic air domain with dimensions of 200 × 200 × 200 mm was constructed as the testing environment, with a time‐varying magnetic field applied on one boundary. The transducer model was established with strict adherence to the actual geometric dimensions, and the piezoelectric effect was confined specifically for BTO ceramics to calculate the output voltage, with material parameters of all components defined using manufacturer‐provided data. To systematically evaluate the influence of key structural parameters on transduction performance, the simulations investigated the system response under various configurations by altering the cantilever beam length, the piezoelectric ceramic length, and the arrangement and quantity of magnetic proof masses. During each simulation, the external magnetic field strength was held constant, and frequency‐domain analysis was employed to extract the system's output characteristics. The simulation results were subsequently log‐transformed and normalized to facilitate comparative analysis between the simulations and experimental measurements, and the processed data were plotted to visualize the performance trends.

### 4.4 Experimental Characterization of the Cantilever‐Based MME Transducer

To evaluate the performance of the cantilever‐based MME transducers, we fabricated a series of prototype devices with various geometries and configurations, which were subsequently secured onto a custom‐designed 3D‐printed holder and positioned centrally within a 1D electromagnetic coil (Figure S16A, link block diagram). To investigate the relationship among excitation frequency, magnetic field strength, and voltage output, a customized LabVIEW program was developed to regulate the stimulation parameters and acquire the corresponding electrical response data. Under fixed magnetic field intensities, the excitation frequency was incrementally swept while the output voltage of the transducer was recorded in real time. To implement the entire testing process, an arbitrary function generator (AFG1062, Tektronix) was used to deliver the programmed electrical signals to a power amplifier (KEPCO), which subsequently boosted the signals and drove the 1D electromagnetic coil to generate the required magnetic excitation. The output of the MME device was captured in real time using an oscilloscope (DHO924, Rigol). All collected data were ultimately transferred through the communication ports to a LabVIEW‐based program on a computer for centralized acquisition and processing. All acquired data were subjected to normalization and log‐transformed to facilitate analysis and the construction of performance distribution maps. To assess the operational stability of the device, the output voltage on the first day of testing (*V*_0*Days*_) was recorded as a reference. Subsequent output voltages (*V_nDays_*) were collected at predetermined time intervals. The stability ratio (*R*_0*Days*_) at each time point was calculated using the following equation:

$$Long-term\;Stability\;Ratio\left(\%\right)=\frac{V_{nDays}}{V_{0Days}}$$

### 4.5 Cell Culture

PC12 cells were obtained from the National Collection of Authenticated Cell Cultures (Chinese Academy of Sciences, China) and revived according to standard protocols. Initial expansion was conducted in T75 culture flasks using complete RPMI‐1640 medium composed of RPMI‐1640 (Hyclone), 10% horse serum (Gibco), 5% fetal bovine serum (FBS, Gibco), and 1% penicillin–streptomycin (Gibco). Under these conditions, cells predominantly maintained a spherical morphology and exhibited a suspended growth pattern, characteristic of the proliferative phase. Once a stable proliferative state was achieved, the culture medium was replaced with differentiation medium consisting of RPMI‐1640 supplemented with 10% FBS and 1% penicillin–streptomycin. Upon switching to the differentiation medium, PC12 cells gradually adopted a polygonal morphology and transitioned to an adherent phenotype. No nerve growth factor (NGF) or other strong differentiation induce is included in the differentiation medium. Therefore, spontaneous neuronal differentiation in the control group remains limited. After stabilization under differentiation conditions, cells were used for subsequent experimental procedures.

### 4.6 Assessment of Cellular Biocompatibility

PDMS sheets subjected to plasma surface treatment were sterilized under UV light for 20 min and subsequently rinsed with PBS. PC12 cells were seeded onto the surface of the materials and cultured under standard conditions for 3, 6, and 9 days. At each time point, culture medium was removed, and the samples were gently washed with PBS. Cell viability was assessed using a live/dead staining kit (Solarbio), the relevant experimental procedures should be carried out in accordance with the official manual. Fluorescence images were acquired using a fluorescence microscope. Calcein‐AM–stained live cells were excited at 490 nm and detected at 515 nm, while PI‐stained dead cells were excited at 535 nm and detected at 617 nm. Cell counts were performed using ImageJ software. The number of live cells (*N_live_*) and dead cells (*N_dead_*) was determined based on fluorescence signals, and the cell viability was calculated using the following equation:

$$Living\;Cells\;Ratio\left(\%\right)=\frac{N_{live}}{N_{live}+N_{dead}}$$

### 4.7 Cellular Electrical Stimulation Experiments

The cellular electrical stimulation experiments consisted of two main parts. In the first part, a prefabricated electrical stimulation device was connected to a standard signal generator (AFG1062, Tektronix) to investigate the effects of different electrical stimulation conditions on PC12 cells. In the second part, the optimized stimulation parameters were applied to stimulate PC12 cells using the MME transducer (Figure S16B, link block diagram) to evaluate the feasibility and stability of the device. For the optimization of electrical stimulation parameters, differentiated PC12 cells were cultured in differentiation medium and seeded onto glass coverslips pre‐treated with poly‐L‐lysine (Solarbio). After ensuring complete cell adhesion, the connected electrical stimulation device was gently positioned at both ends of the coverslip. Electrical stimulation was then applied under parameters set according to specific experimental requirements.

For the feasibility and stability evaluation using the MME transducer, the wireless electrical stimulation device was similarly positioned at both ends of the coverslip and placed within a magnetic field generator. Electrical stimulation was performed using the confirmed optimal parameters. Upon completion of the stimulation protocols, the cells were collected and processed for subsequent analyses.

### 4.8 Morphological Analysis of PC12 Cells

Following electrical stimulation—either via the standard waveform generator or the cantilever‐based stimulation device—cells were imaged using a phase‐contrast microscope. For each coverslip, five random fields of view were selected for imaging. Quantitative morphological analysis was performed using ImageJ software. Key parameters included the total number of cells per field (*N_cell_*), the number of neurite‐bearing cells (*N_synapse_*), and the maximum neurite length per cell (*L_synapse_*). The proportion of neurite‐bearing cells was calculated as:

$$Synapse\;Expression\;Ratio\left(\%\right)=\frac{N_{synapse}}{N_{cell}}$$

All data were statistically analyzed and plotted to evaluate the effects of different stimulation conditions on neuronal differentiation and morphological changes.

### 4.9 Immunofluorescence Staining

Following electrical stimulation, PC12 cells were fixed with 4% paraformaldehyde (PFA, Biosharp) at room temperature for 30 min. After fixation, cells were incubated with a blocking solution consisting of 5% bovine serum albumin (BSA, Solarbio) and 0.1% Triton X‐100 in PBS at room temperature for 10 min. Subsequently, cells were incubated overnight at 4°C with a primary antibody against βIII‐tubulin (rabbit anti‐βIII‐tubulin, Abcam, 1:500, diluted in blocking solution). After three washes with PBS, cells were incubated at room temperature for 1 h with an Alexa Fluor 488‐conjugated goat anti‐rabbit secondary antibody (1:600, diluted in blocking solution). Nuclear staining was performed by incubating cells with Hoechst 33342 (Solarbio, 1:600, prepared in PBS) at room temperature for 10 min, followed by PBS washes. Finally, samples were mounted with 75% (v/v) glycerol prepared in distilled water and imaged using a confocal laser scanning microscope (Leica TCS SP8 STED 3X).

### 4.10 Quantitative Real‐Time PCR (qPCR)

PC12 cells subjected to electrical stimulation were harvested using TRI Reagent (1 mL per sample, Sigma‐Aldrich) and lysed at room temperature for 10 min. The lysates were transferred to RNase‐free tubes and centrifuged at 12 000 rpm for 20 min at room temperature. The supernatant was transferred to a new tube, mixed with 200 µL chloroform, vigorously vortexed, and incubated at room temperature for 10 min. Following centrifugation at 12 000 rpm for 10 min, the aqueous phase was collected, and RNA was precipitated by adding 500 µL isopropanol. After incubation at room temperature for 20 min, samples were centrifuged at 12 000 rpm for 10 min, and the RNA pellet was washed with 75% ethanol (prepared with DEPC‐treated water) and centrifuged at 8,000 rpm for 10 min. The resulting RNA pellet was air‐dried and dissolved in 20 µL DEPC‐treated water for subsequent analyses. For cDNA synthesis, 1 µg of total RNA was reverse transcribed using the RevertAid First Strand cDNA Synthesis Kit (Thermo Scientific) according to the manufacturer's instructions. Quantitative real‐time PCR (qRT‐PCR) was performed using TB Green Premix Ex Taq II (TaKaRa) on a CFX Connect Real‐Time PCR Detection System (Bio‐Rad) following the manufacturer's protocols. All primer sequence information is detailed in Table S2. Relative gene expression levels were calculated using the ΔΔCt method, normalized to GAPDH, with the following formulas:

$$Q_{gene}^{Ctrl}=2^{-\left[\left(Ct_{gene}^{Ctrl}-\bar{Ct_{GAPDH}^{Ctrl}}\right)-\left.\bar{Ct_{gene}^{Ctrl}-\bar{Ct_{GAPDH}^{Ctrl}}}\right)\right]}$$

$$Q_{gene}^{ES}=2^{-\left[\left(Ct_{gene}^{ES}-\bar{Ct_{GAPDH}^{ES}}\right)-\left.\bar{Ct_{gene}^{Ctrl}-\bar{Ct_{GAPDH}^{Ctrl}}}\right)\right]}$$

### 4.11 Establishment of the Animal Model

Male Sprague‐Dawley (SD) rats weighing approximately 300 g were obtained from the Animal Center of Xi'an Jiaotong University. All procedures related to animal housing, surgical interventions, and handling strictly adhered to institutional ethical guidelines approved by the Ethics Committee of Xi'an Jiaotong University (approval number: to be provided). All biomedical waste generated from animal experiments was collected and safely disposed of by certified biosafety organizations. Prior to surgery, all surgical instruments were thoroughly sterilized, and the rats were anesthetized using inhalational anesthesia. Following hair removal and disinfection, a longitudinal skin incision of approximately 15 mm was made along the lateral aspect of the thigh. Under sterile conditions, the muscle layers were bluntly dissected to expose the sciatic nerve. After identifying the nerve, a standardized crush injury was applied using a needle holder, maintaining consistent compression for 10 s. In the wireless electrical stimulation group and the wireless‐stimulator control group, a wireless electrical stimulation device was implanted into the thigh cavity. The injured sciatic nerve was gently guided into the flexible fixation conduit within the device, ensuring that the crushed segment was precisely positioned between the embedded gold electrodes and that the electrode contact points were accurately aligned with both the proximal and distal ends of the injury site. Following device implantation (whereas in the spontaneous regeneration group, no further intervention was performed post‐injury), the muscle and skin layers were sutured, and the incision site was disinfected with iodine solution. The rats were subsequently returned to their cages for recovery and subsequent experimental procedures.

### 4.12 In Vivo Treatment of the Animal Model

Following surgical recovery, rats were subjected to stimulation and evaluation protocols based on the grouping and treatment regimens outlined. For animals in the Stimulated Wireless Device group, magnetic excitation was performed by placing the rats within a 1D coil system. Magnetic stimulation was applied in accordance with the parameters listed above: 60 Hz frequency, 2 Oe field intensity, and 10 min per session, administered every other day over a 4‐week period (Figure S16B, link block diagram).

### 4.13 Behavioral Assessment of Sciatic Nerve Function

Assessment of motor function recovery was performed by analyzing gait patterns in rats across experimental groups. Animals were first placed in a transparent locomotion track (100 × 100 × 600 mm^3^) for habituation, with each session lasting no less than 30 min to allow sufficient environmental acclimatization. Following habituation, the track was thoroughly cleaned to eliminate residual odors or excreta that might interfere with behavioral performance. For gait acquisition, the plantar surfaces of both hind paws were coated with non‐toxic ink, and the animals were allowed to traverse the runway freely from one end to the other. Upon completion, the animals were immediately returned to their home cages. The following footprint parameters were measured for both the normal (N) and experimental (E) limbs: print length (PL), toe spread (TS, defined as the distance between the first and fifth toes), and intermediate toe spread (IT, defined as the distance between the second and fourth toes). Based on these measurements, the sciatic functional index (SFI) was calculated according to the formula described by Bain et al. [67], providing a quantitative assessment of sciatic nerve function:

$$SFI=\frac{109.5*\left(ETS-NTS\right)}{NTS}\;-\frac{38.3*\left(EPL-NPL\right)}{NPL}+\frac{13.3*\left(EIT-NIT\right)}{NIT}-8.8$$

### 4.14 Preparation of Frozen Tissue Sections

Rats were euthanized in accordance with institutional animal care and ethical guidelines. After sacrifice, the gastrocnemius muscle and the injured sciatic nerve segment are carefully harvested for subsequent tissue evaluation. For gastrocnemius muscle processing, samples were rinsed with phosphate‐buffered saline (PBS) and subsequently fixed in 4% paraformaldehyde (PFA) at room temperature for 4 h. Following fixation, the tissues were dehydrated overnight at 4°C in a 30% (w/v) sucrose solution prepared in PBS and then rapidly frozen in isopentane cooled with liquid nitrogen. The frozen samples were then placed at −20°C to allow the evaporation of residual isopentane before being embedded in optimal cutting temperature (OCT) compound (SAKURA) for cry‐sectioning. Similarly, the extracted sciatic nerve samples are promptly processed for fixation and section preparation to preserve tissue integrity for the subsequent histological and immunofluorescence analyses. Tissues were washed with PBS and fixed in 4% PFA at 4°C overnight. After fixation, the tissues were sequentially dehydrated in 20% and 30% (w/v) sucrose solutions in PBS at 4°C overnight. The dehydrated tissues were then embedded in OCT compound for cry‐sectioning. Embedded samples were sectioned using a cryostat microtome (Leica) with the chamber and blade temperatures maintained at approximately −23°C. Sections of the gastrocnemius muscle were cut at a thickness of 12 µm, while sections of the sciatic nerve were prepared at 8 µm. All sections were mounted on adhesive microscope slides and stored at −20°C until further use.

### 4.15 Masson's Trichrome Staining of Muscle Tissue

Frozen muscle sections were equilibrated to room temperature for 20 min prior to staining. Masson's trichrome staining was performed using a commercial kit (Solarbio) following the manufacturer's instructions. After staining, the sections were imaged using a conventional light microscope, and quantitative analysis of muscle fiber cross‐sectional area (*S_muscle_*) was performed using ImageJ.

### 4.16 Toluidine Blue Staining of Sciatic Nerve Tissue

Frozen sciatic nerve sections were equilibrated to room temperature for 20 min prior to staining. The sections were then stained with toluidine blue solution (Solarbio) following the manufacturer's instructions. Bright‐field images were acquired using a conventional optical microscope. Quantitative analysis was performed using ImageJ, where the number of toluidine blue‐positive neuronal cells within the sciatic nerve cross‐section was counted (*N_neurone_*), and the corresponding neural fiber area was measured (*S_fiber_*). The average neuronal cell density was calculated as:

$$Neurone\;Expression\;Ratio\left(\%\right)=\frac{N_{neurone}}{S_{fiber}}$$

### 4.17 Tissue Immunofluorescence Staining

Frozen tissue sections were equilibrated to room temperature for 20 min prior to staining. Following equilibration, sections were blocked with a BSA blocking buffer at room temperature for 30 min. Primary antibodies, including βIII‐Tubulin (rabbit anti‐βIII‐Tubulin, Abcam, 1:400, diluted in BSA buffer) and S100 (rabbit anti‐S100, Abcam, 1:400, diluted in BSA buffer), were applied and incubated overnight at 4°C.

After primary antibody incubation, sections were washed thoroughly with PBS and incubated with secondary antibody (Alexa Fluor 488‐conjugated goat anti‐rabbit, 1:600, diluted in BSA buffer) for 1 h at room temperature in the dark. Nuclei were counterstained with Hoechst 33342 (1:600 in PBS) for 10 min at room temperature in the dark, followed by PBS washing. Slides were mounted using 75% glycerol and imaged using a confocal laser scanning microscope. Quantitative analysis was performed using ImageJ. The area of specific protein immunoreactivity (*S_protein_*) and total nuclear area (*S*_*nucleus* _) were measured to assess relative protein expression, calculated as:

$$Protein\;Expression\;Ratio\left(\%\right)=\frac{S_{protein}}{S_{nucleus\;}}$$

### 4.18 Image Acquisition and Analysis

All images were captured using a Nikon Z6II camera. Confocal microscopy images were processed with the proprietary Leica software suite. Quantitative image analysis was performed using ImageJ, as described in the Methods section. Graphs and statistical plots were generated using GraphPad Prism. Statistical significance was assessed using two‐way ANOVA implemented within the software, and all significance levels and corresponding annotations are detailed in the Figure legends.

### 4.19 Ethical Approval

This research compiles all relevant ethical regulations. The study protocol was approved by the Institutional Review Board of the School of Life and Science Technology of Xi'an Jiaotong University (No. 2025‐1).

## Author Contributions

Yijing Wang, Zhilu Ye, and Xiaohui Zhang conceived the idea and led research efforts. Yijing Wang and Jingyu Tian conducted numerical simulations. Yijing Wang and Jingen Wu performed the device fabrication and electrical experiment. Yijing Wang and Cuiling Zhang led animal studies and performed surgeries. Yijing Wu and Zhe Xu contributed to biocompatibility and cellular experiments. Yijing Wang, Zhilu Ye, Ming Liu, and Xiaohui Zhang wrote the manuscript.

## Funding

This work was financially supported by the National Key Research and Development Program of China (2022YFB3206805HZ), the supporting project (GXKJXM20230186), and the National Natural Science Foundation of China (Grant No. 62401454).

## Conflicts of Interest

The authors declare no conflict of interest.

## Supporting information




**Supporting File**: advs75833‐sup‐0001‐SuppMat.docx.

## Data Availability

The data that support the findings of this study are available from the corresponding author upon reasonable request.
