# Supplementary material for: A Millimeter‐Scale Implantable Magneto–Mechano–Electric Transducer Based on BTO Piezoelectric Ceramics for Remote Wireless Electrical Stimulation of Injured Sciatic Nerves
Source: Adv Sci (Weinh). 2026 May 25:e75833. Online ahead of print. doi: 10.1002/advs.75833 (PMC13335879; doi:10.1002/advs.75833)
Supplement: Supplementary file 1 — Supporting File: advs75833‐sup‐0001‐SuppMat.docx. [file ADVS-9999-e75833-s001.docx]

**A Millimeter-Scale Implantable Magneto–Mechano–Electric Transducer Based on BTO Piezoelectric Ceramics for Remote Wireless Electrical Stimulation of Injured Sciatic Nerves**

Yijing Wang *et al.*

*Corresponding author. E-mail: [xiaohuizhang@mail.xjtu.edu.cn](mailto:xiaohuizhang@xjtu.edu.cn); [zhiluye@xjtu.edu.cn](mailto:zhiluye@xjtu.edu.cn); [mingliu@xjtu.edu.cn](mailto:mingliu@xjtu.edu.cn);

**This PDF file includes:**

Figs. S1 to S17

Tables S1 and S2

**
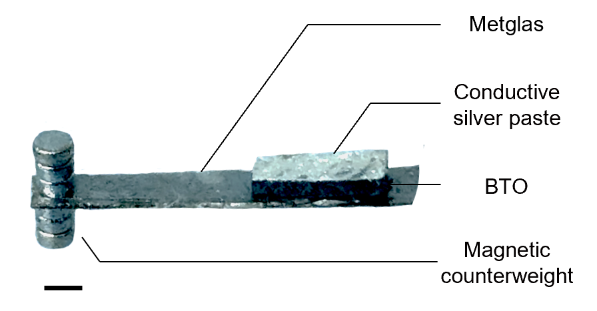
**

Fig. S1. Photograph of the cantilever-based MME device. in the photo, scale bar, 1mm.

**
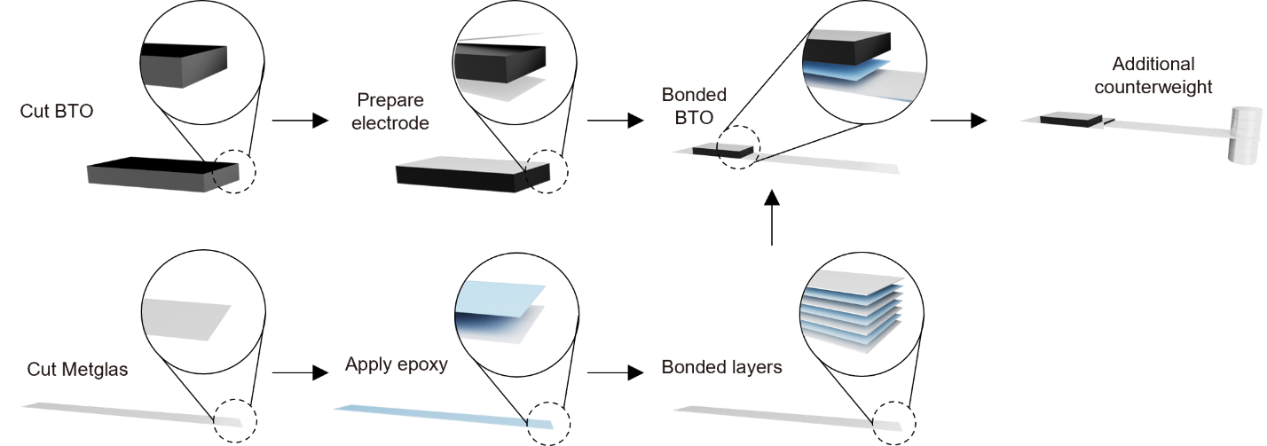
**

Fig. S2. Schematic diagram illustrates the construction of the cantilever-based MME device.


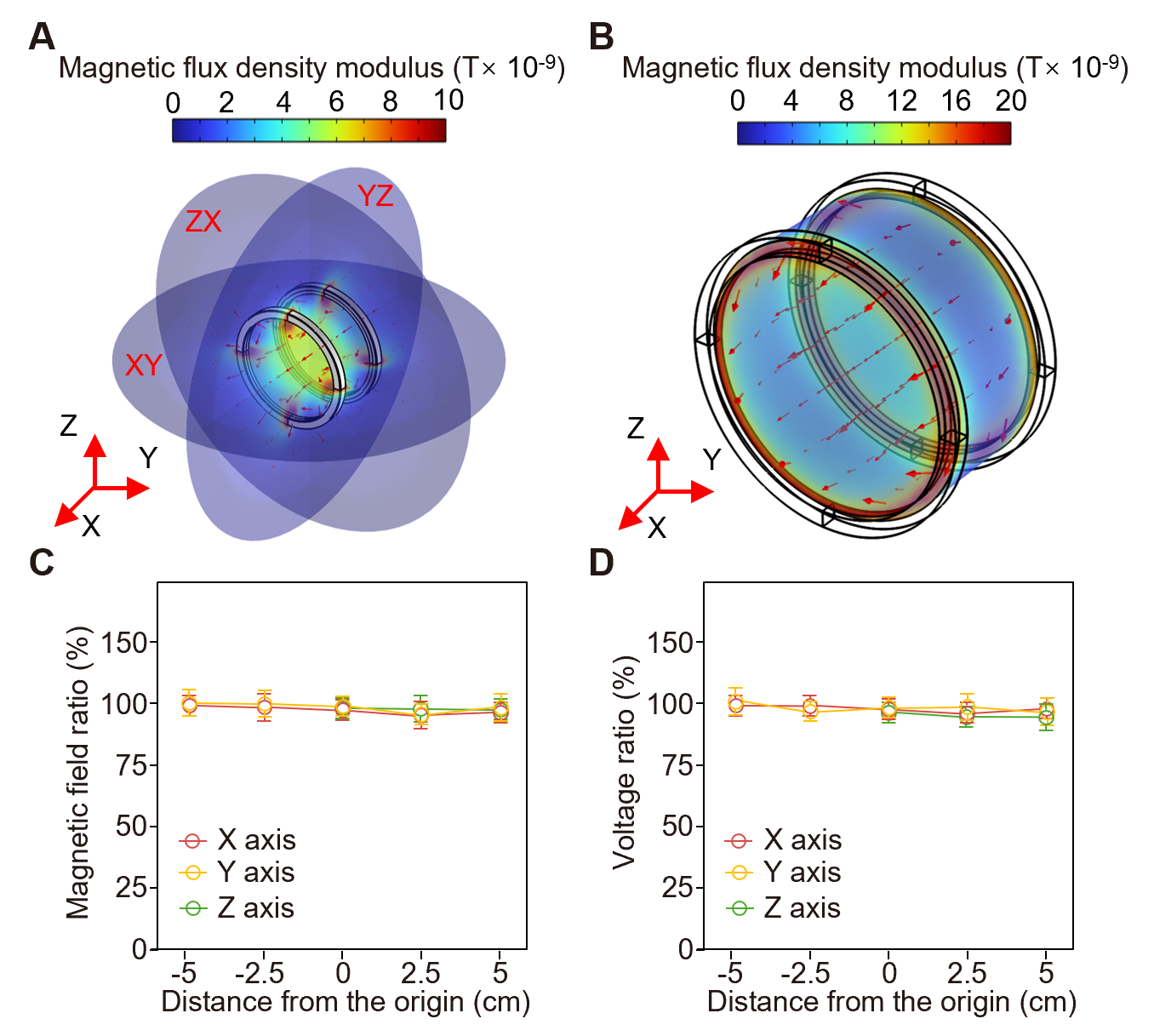


**Fig. S3. Output performance of the implanted device within the Helmholtz coil system.** (**A**) Simulated distributions of magnetic field intensity in the XY, YZ, and ZX planes. (**B**) Simulated spatial distribution of magnetic field intensity within the coil. (**C**) Statistical analysis of measured magnetic field intensity at different positions along with the positive and negative directions of the X-axis, Y-axis, and the positive directions of the Z-axis. (**D**) Statistical analysis of the output performance of the implanted device at different positions along the positive and negative directions of the X-axis, Y-axis, and the positive directions of the Z-axis

**
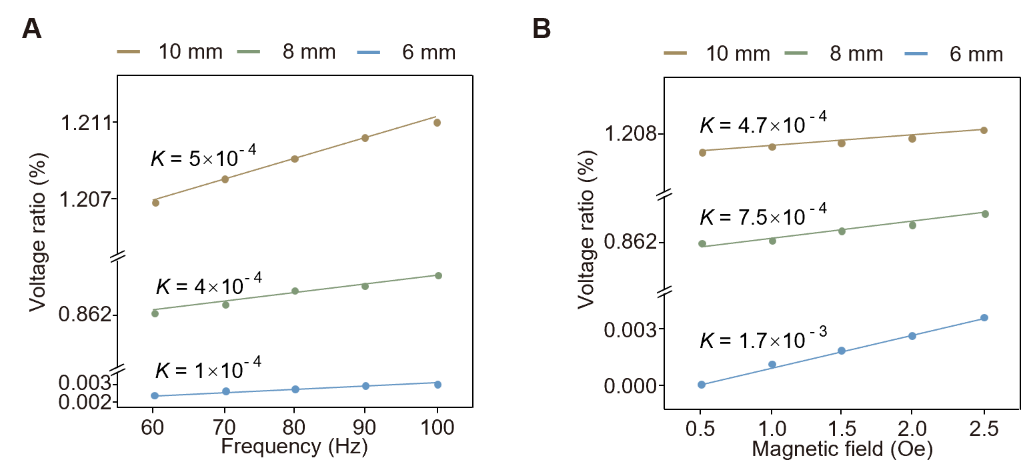
**

Fig. S4. Length optimization of the cantilever beam in the MME system. (A) Linear plots comparing simulated (solid lines) and experimental (dots) output voltages at different frequencies (60–100 Hz) for cantilevers of varying lengths (6 mm, 8 mm, and 10 mm) under a fixed magnetic field strength of 2 Oe (BTO ceramic length: 2 mm; counterweight mode: single weight). (B) Linear plots comparing simulated (solid lines) and experimental (dots) output voltages at different magnetic field strength (0.5–2.5 Oe) for cantilevers of varying lengths (6 mm, 8 mm, and 10 mm) under a fixed frequency of 60 Hz (BTO ceramic length: 2 mm; counterweight mode: single weight).

**
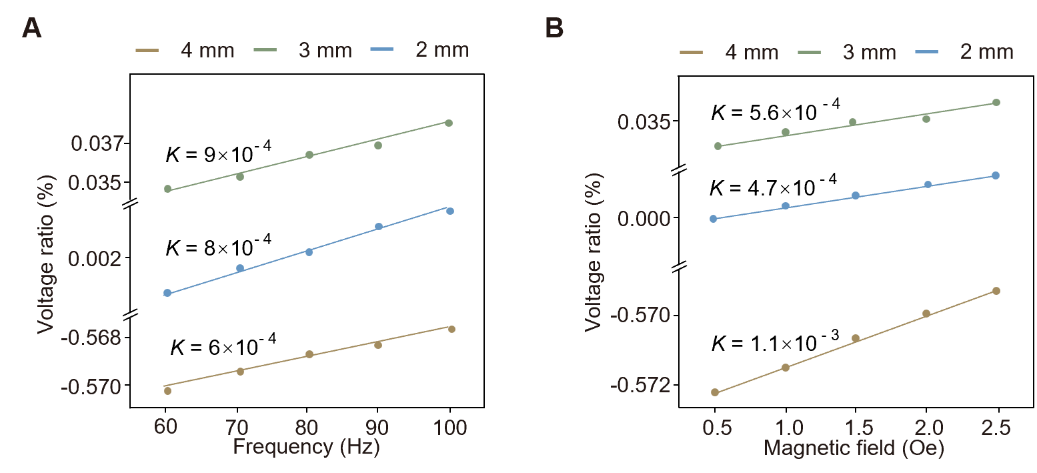
**

Fig. S5. Length optimization of BTO ceramics in the MME system. (A) Linear plots comparing simulated (solid lines) and experimental (dots) output voltages at different frequencies (60–100 Hz) for BTO ceramics of varying lengths (2 mm, 3 mm, and 4 mm) under a fixed magnetic field strength of 2 Oe (cantilevers length: 10 mm; counterweight mode: single weight). (B) Linear plots comparing simulated (solid lines) and experimental (dots) output voltages at different magnetic field strength (0.5–2.5 Oe) for BTO ceramics of varying lengths (2 mm, 3 mm, and 4 mm) under a fixed frequency of 60 Hz (cantilevers length: 10 mm; counterweight mode: single weight).


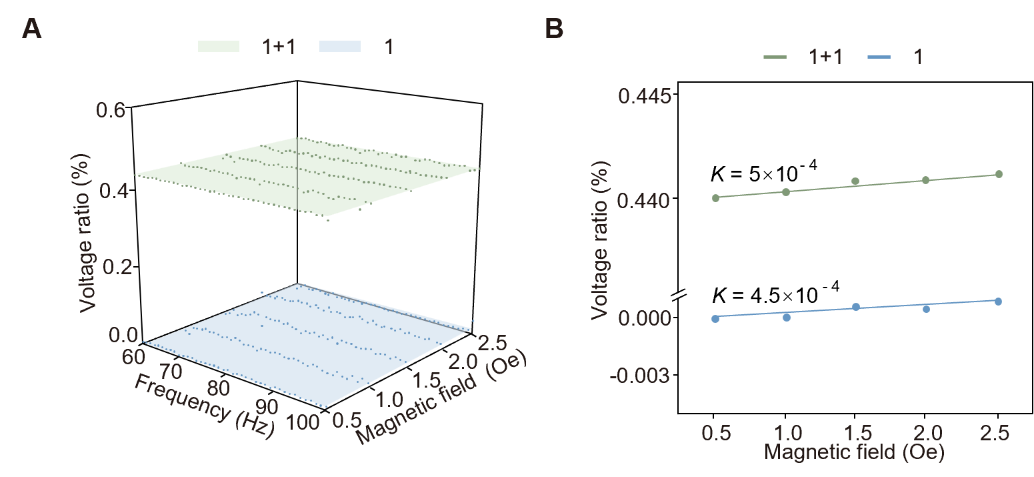


Fig. S6. Optimization of the magnetic counterweight mode (1 & 1+1 mode) in the MME system. (A) 3D comparative plot of simulation results (surface) and experimental measurements (dots) output voltages at different frequencies (60–100 Hz) and different magnetic field strength (0.5-2.5Oe) for 1 mode and 1+1 mode of magnetic counterweight (cantilevers length: 10 mm; BTO ceramic length: 3 mm). (B) Linear plots comparing simulated (solid lines) and experimental (dots) output voltages at different frequencies (60–100 Hz) and different magnetic field strength (0.5-2.5Oe) for 1 mode and 1+1 mode of magnetic counterweight (cantilevers length: 10 mm; BTO ceramic length: 3 mm).

**
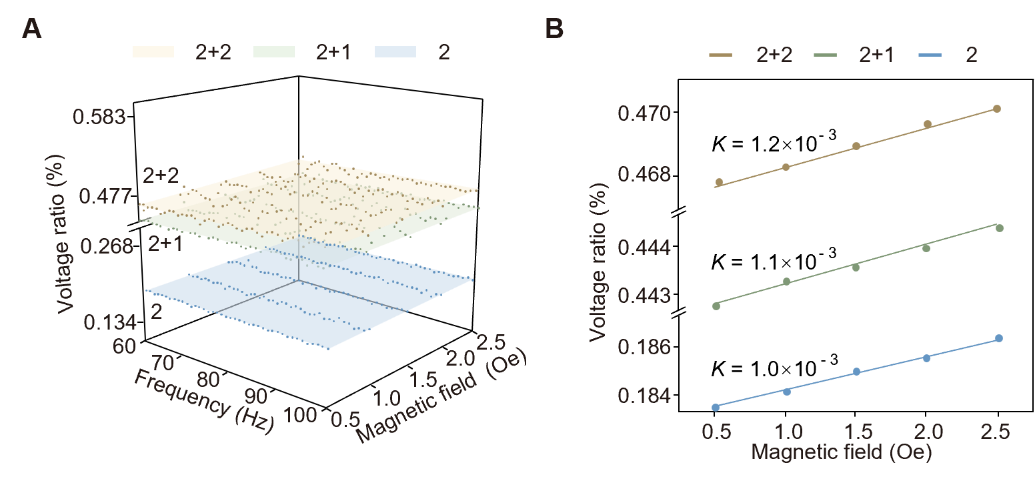
**

Fig. S7. Optimization of the magnetic counterweight mode (2, 2+1 & 2+2 mode) in the MME system. (A) 3D comparative plot of simulation results (surface) and experimental measurements (dots) output voltages at different frequencies (60–100 Hz) and different magnetic field strength (0.5-2.5Oe) for 2 mode, 2+1 mode and 2+2 mode of magnetic counterweight (cantilevers length: 10 mm; BTO ceramic length: 3 mm). (B) Linear plots comparing simulated (solid lines) and experimental (dots) output voltages at different frequencies (60–100 Hz) and different magnetic field strength (0.5-2.5Oe) for 2 mode, 2+1 mode and 2+2 mode of magnetic counterweight (cantilevers length: 10 mm; BTO ceramic length: 3 mm).

**
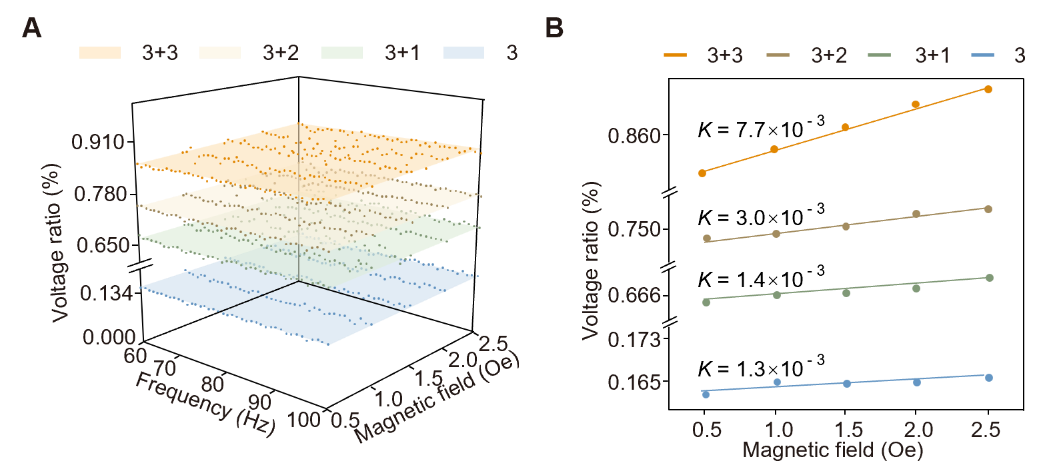
**

Fig. S8. Optimization of the magnetic counterweight mode (3, 3+1, 3+2 & 3+3 mode) in the MME system. (A) 3D comparative plot of simulation results (surface) and experimental measurements (dots) output voltages at different frequencies (60–100 Hz) and different magnetic field strength (0.5-2.5Oe) for 3 mode, 3+1 mode, 3+2 mode and 3+3 mode of magnetic counterweight (cantilevers length: 10 mm; BTO ceramic length: 3 mm). (B) Linear plots comparing simulated (solid lines) and experimental (dots) output voltages at different frequencies (60–100 Hz) and different magnetic field strength (0.5-2.5Oe) for 3 mode, 3+1 mode, 3+2 mode and 3+3 mode of magnetic counterweight (cantilevers length: 10 mm; BTO ceramic length: 3 mm).


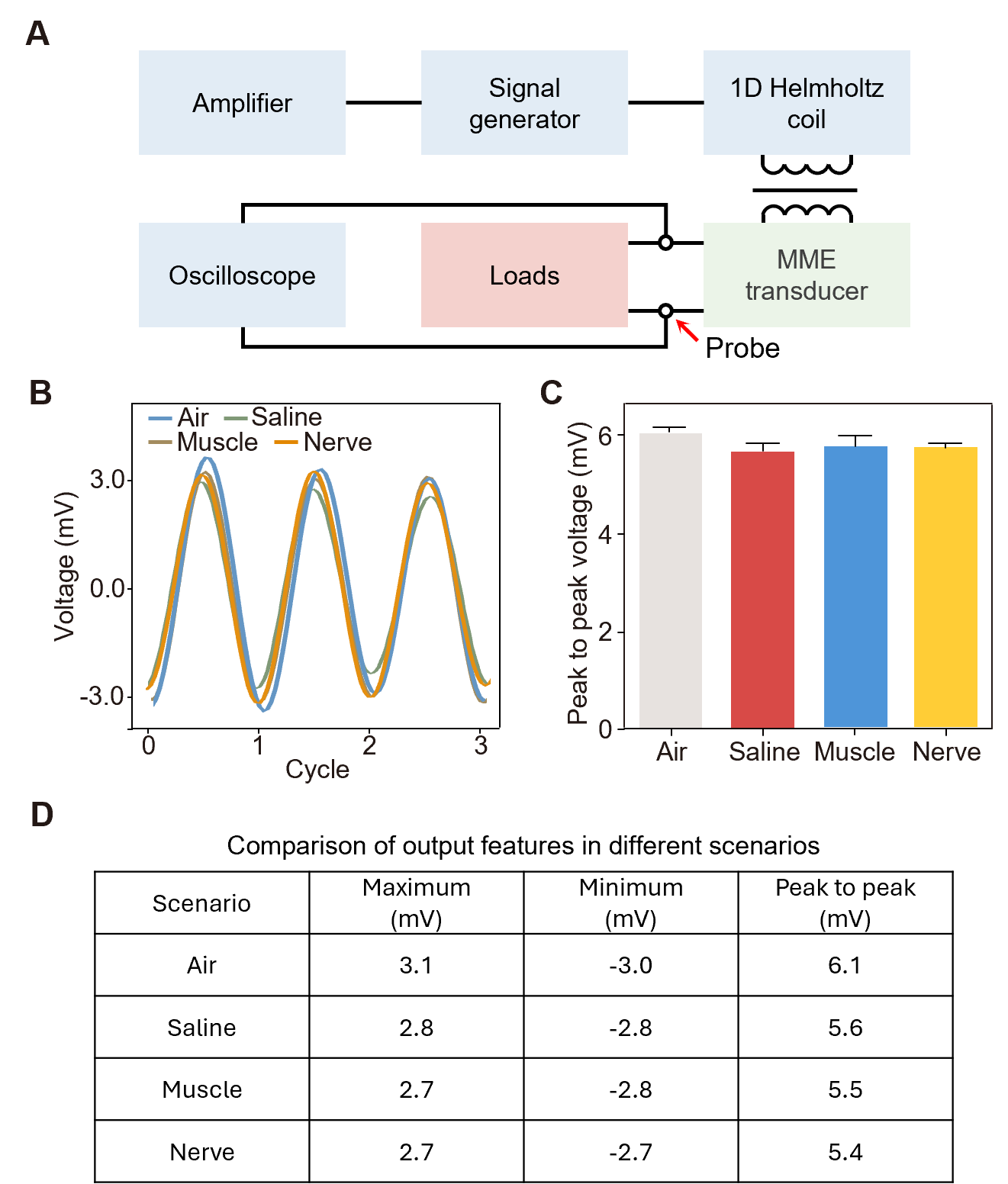


**Fig. S9. Voltage output evaluation under realistic scenarios with biological loads. (A)** Structural block diagram of the testing loading system for the cantilever-based MME energy conversion device. (**B**) The output waveforms over a fixed period are presented in four distinct loads, including air, physiological saline, muscle tissue, and neural tissue. (**C**) Summarizes the corresponding peak-to-peak output value across these loads in a bar chart format. (**D**) Provides detailed waveform characteristics under each load’s condition.


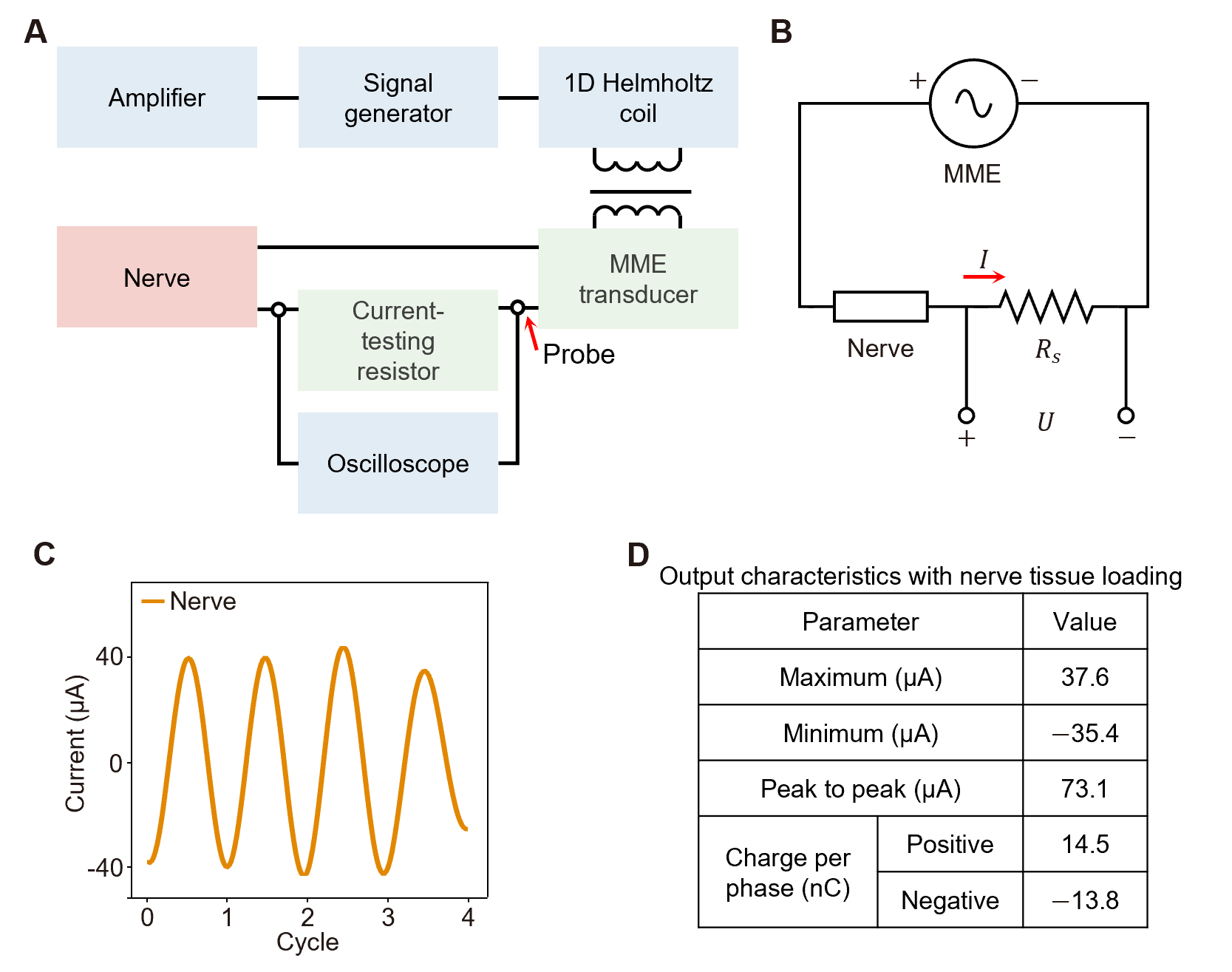


**Fig. S10. Current output evaluation under realistic scenarios with nerve loads. (A)** Structural block diagram of the testing loading system for the cantilever-based MME energy conversion device. **(B)** Internal circuit diagram of the current measurement setup for the testing loading system. **(C)** The output waveforms over a fixed period are presented in neural tissue. **(D)** Provides detailed waveform characteristics under nerve load’s condition. The relevant calculation formulas used for the data presented in the table are as follows: The current was calculated according to $I(t)=U(t)/R_{s}$, where $I$ represents the current magnitude, $U$ represents the measured voltage magnitude, and $Rs$ represents the measured resistance value. The charge per phase was calculated according to $Q=\int I(t)dt$, where $Q$ represents the charge delivered per phase, $I$ represents the current magnitude, and $t$ represents the corresponding time. The current density was calculated according to$J(t)=I(t)/A$, where $J$ represents the current density, $I$ represents the current magnitude, and $A$ represents the contact area.

**
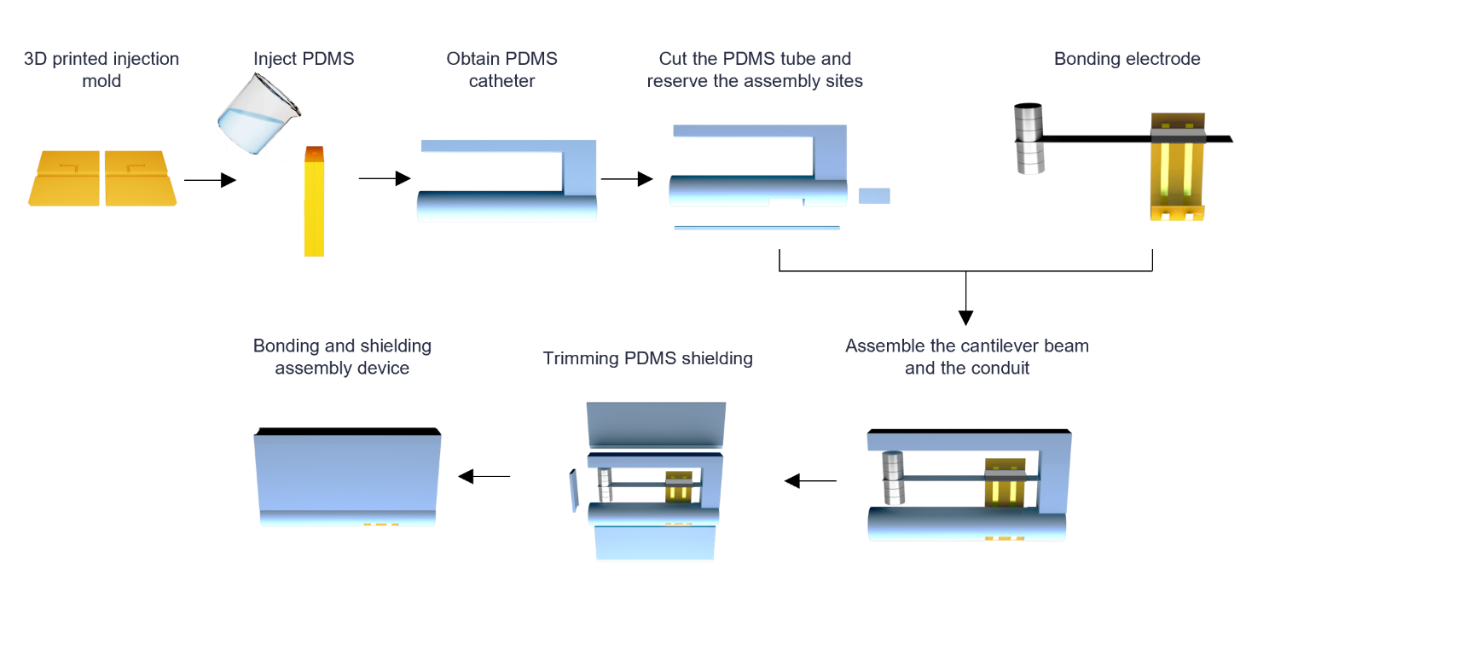
**

Fig. S11. Schematic diagram of the assembly process for the wireless electrostimulation device.


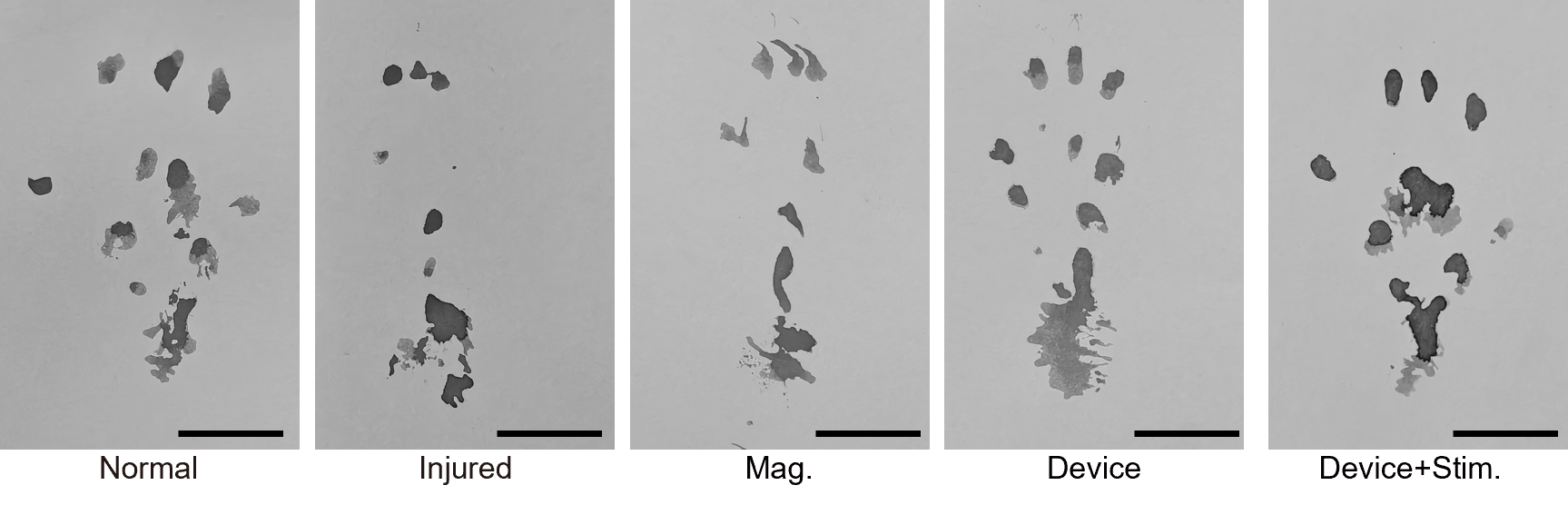


**Fig. S12.** Representative footprint images showing hindlimb locomotor function in rats from the normal group (Normal), injury group (Injured), magnetic-field-only group (Mag.), device-only group (Device), and device-stimulation group (Device+Stim.) after 4 weeks of nerve repair. Scale bars, 10 mm

**
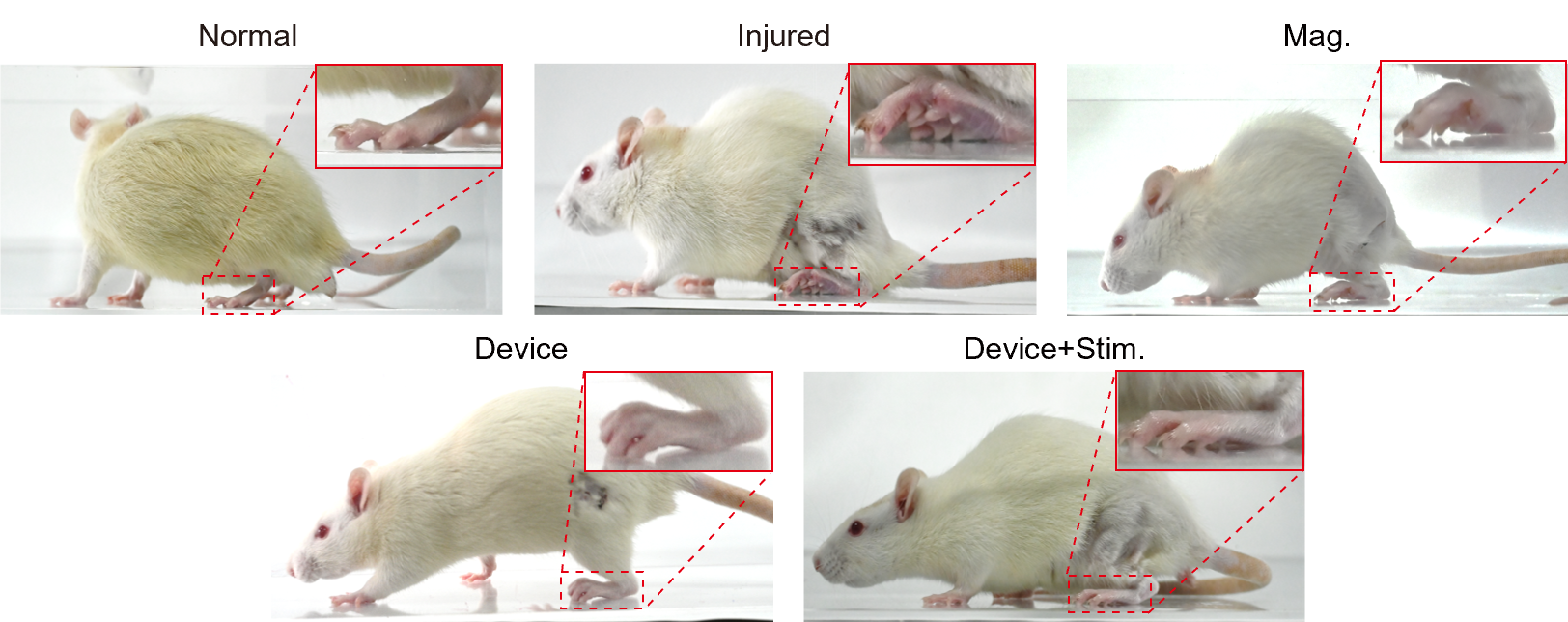
**

Fig. S13. Representative images of hindlimb motor function in rats from the normal group (Normal), injury group (Injured), magnetic-field-only group (Mag.), device-only group (Device), and device-stimulation group (Device+Stim.) following 4 weeks of nerve repair.


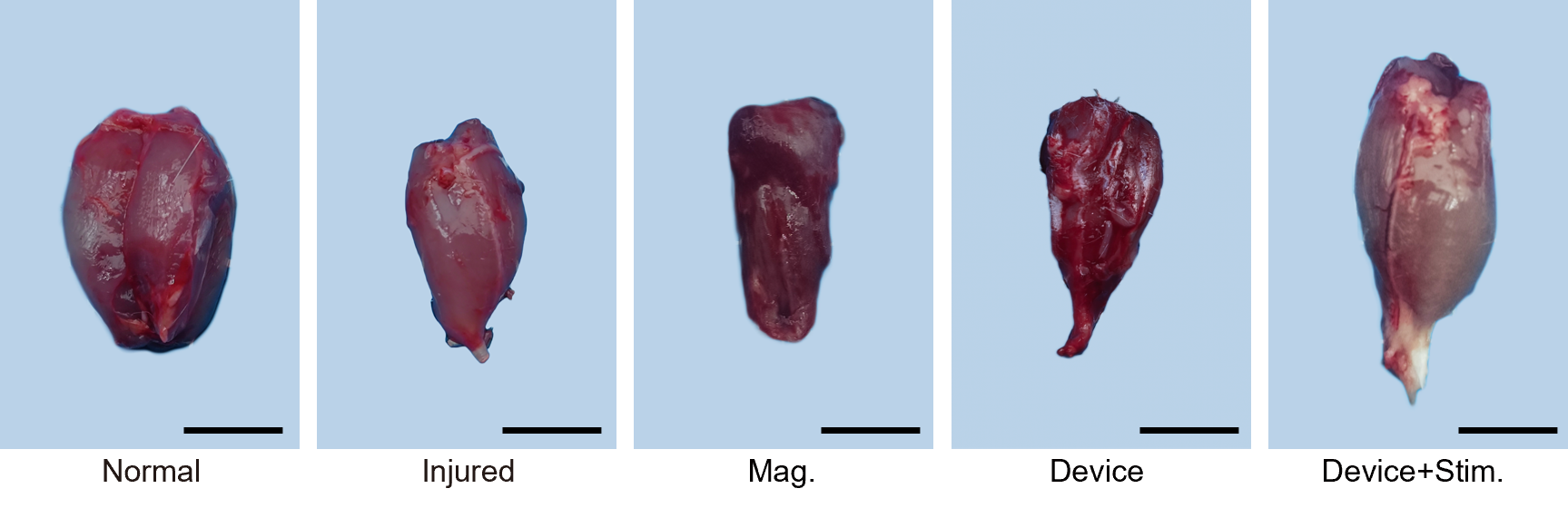


**Fig. S14.** Representative images of gastrocnemius muscle tissue from rats in the normal group (Normal), injury group (Injured), magnetic-field-only group (Mag.), device-only group (Device), and device-stimulation group (Device+Stim.) after 4 weeks of nerve repair. Scale bars, 1 cm.


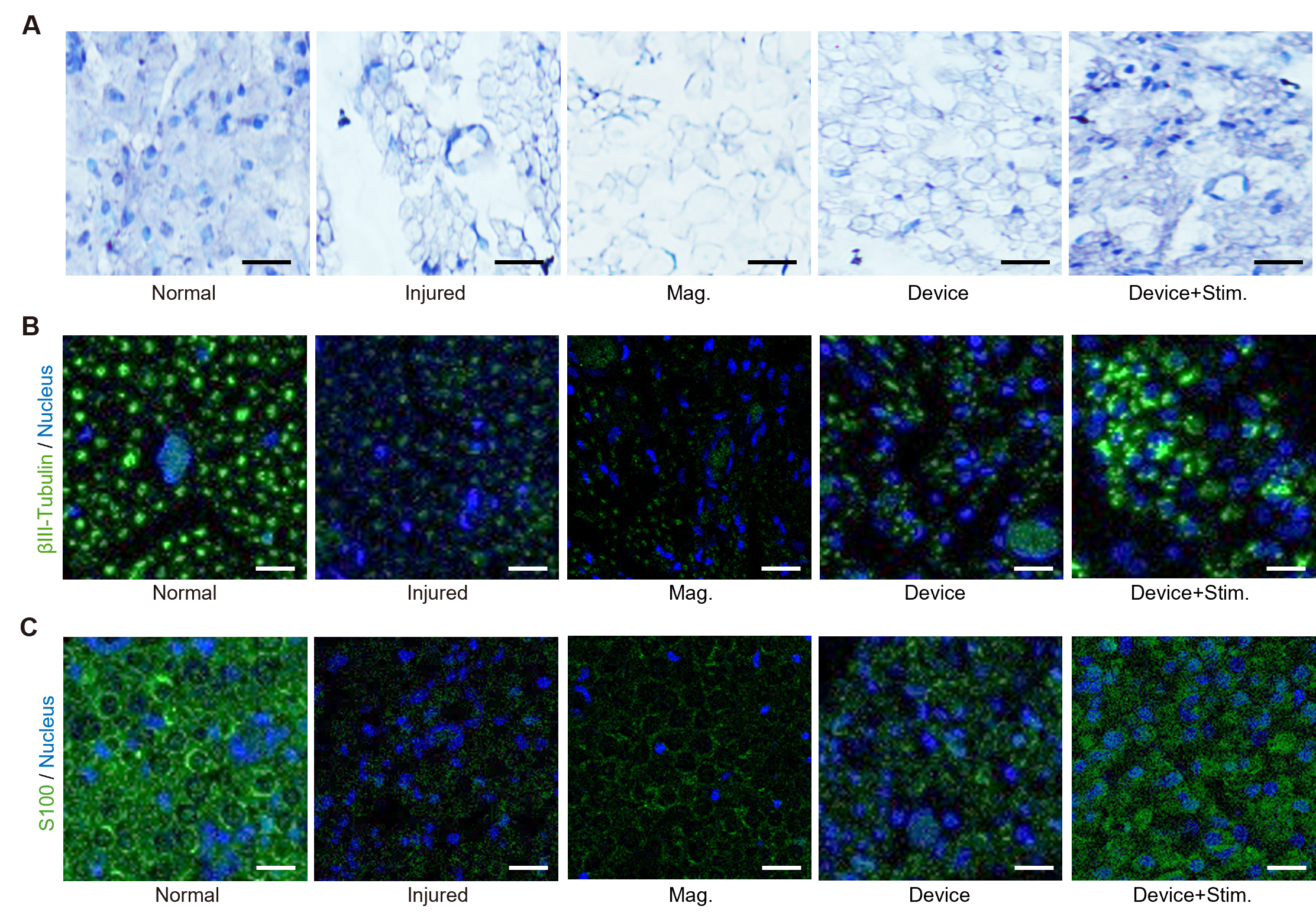


Fig. S15. Histological staining enlarged images illustrate the restoration of sciatic nerve injury in a rat model facilitated by a wireless electrical stimulation device. (A) Representative Toluidine Blue staining images of sciatic nerve cross-sections in the normal group (Normal), injury group (Injured), magnetic-field-only group (Mag.), device-only group (Device), and device-stimulation group (Device+Stim.). (B) Representative immunofluorescence staining of S100 protein in sciatic nerve tissue in each group. (blue: Hoechst; green: S100). (C) Representative immunofluorescence staining of βIII-Tubulin in sciatic nerve sections in each group. (blue: Hoechst; green: βIII-Tubulin). Scale bars, 50 μm.

**
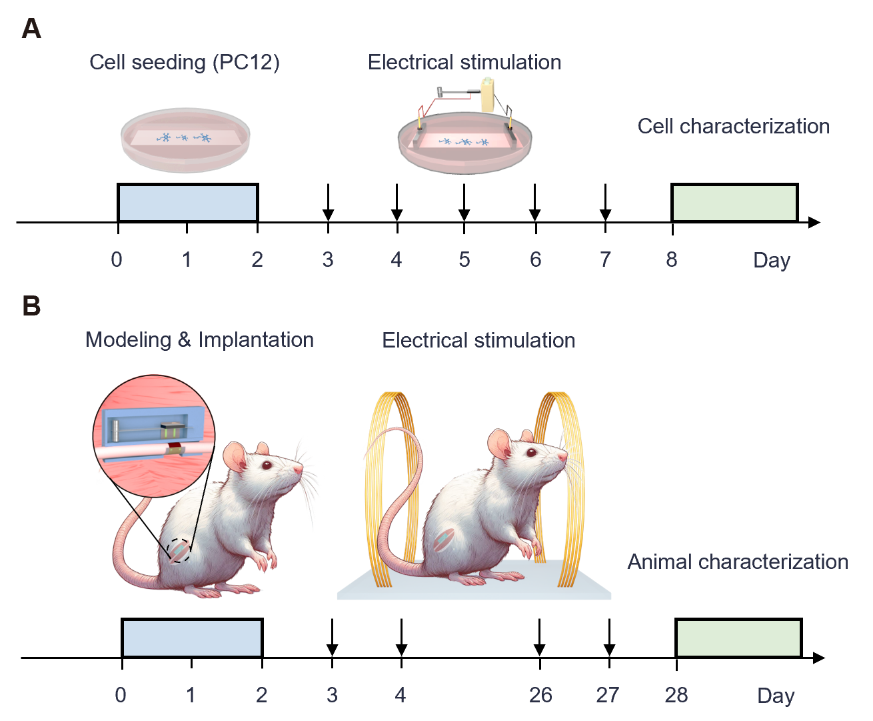
**

Fig. S16. Schematic overview of cell and animal experimental procedures. (A) Flowchart illustrates the experimental procedure for cellular electrical stimulation using the cantilever-based MME energy conversion device. (B) Flowchart depicting the experimental procedure for sciatic nerve injury repair in a rat model using the wireless electrostimulation therapeutic device.

**
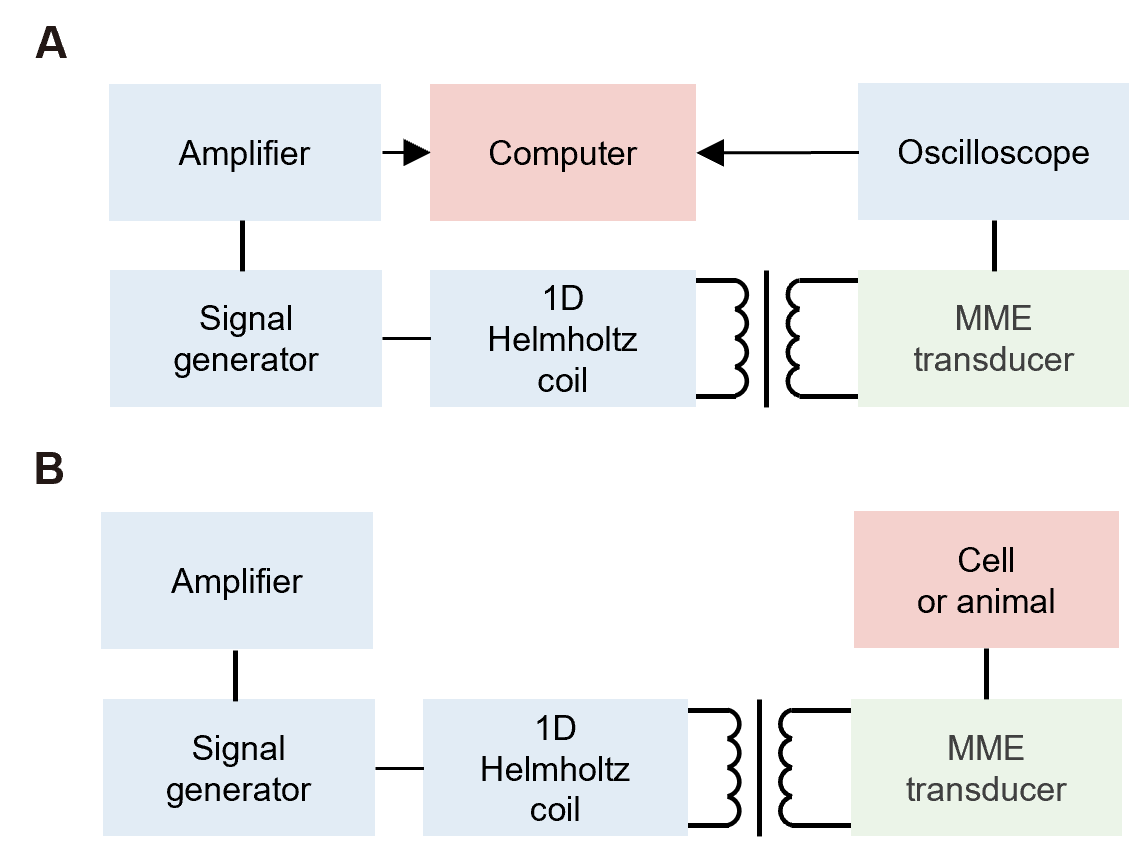
**

Fig. S17. Block diagrams of testing and stimulation systems. (A) Structural block diagram of the testing system for the cantilever-based MME energy conversion device. (B) Structural block diagram for in vitro cell stimulation or in vivo stimulation of animal models using the cantilever-based MME energy conversion device.

**Table S1.** Comparison between the developed implantable device and existing implantable device/ material based on the piezoelectric effect.

| Ref. | Device Size  (mm) | IC | Stimulation Modality | Stimulation Distance  (mm) | Stimulus Frequency  (Hz) | Output Voltage | Application Scenarios |
| --- | --- | --- | --- | --- | --- | --- | --- |
| Piezoelectric Device | | | | | | | |
| [1] | 13.6×7.3×2 | Yes | ultrasound | 20 | 1 or 3M | ~0.6W | nervous system |
| [2] | 13.5×9.6×2.1 | No | ultrasound | 30 | 1M | ~280uW | nervous system |
| [3] | 12×44 | Yes | ultrasound | 25 | 3.3M | ~2V | visual system |
| [4] | 4×7.8 | Yes | ultrasound | 30 | 3.5-4.5G | 1V | - |
| [5] | 6×12 (φ) | Yes | ultrasound | 8 | 1M | 5.95W | nervous system |
| [6] | 30×5 | Yes | ultrasound | - | 1M | 417mV | nervous system |
| [7] | 20×20 | No | pressure | - | - | 8V | cardiac system |
| [8] | 3×2.15×14.8 | Yes | ME field | 25 | 350K | 8V | nervous system |
| [9] | 5×9 | Yes | ME field | 30 | 250K | 1.5mA | nervous system |
| Piezoelectric Material | | | | | | | |
| [10] | 15×15 | No | ultrasound | - | 100K | 0.9mA | nervous system |
| [11] | 2(φ) | No | ultrasound | - | 1M | ~30uA | nervous system |
| [12] | 10×2(φ) | No | ultrasound | 25 | 1M | ~60mV | nervous system |
| [13] | - | No | ultrasound | - | 1.5M | 6.7uA | skeleton system |
| [14] | - | No | ultrasound | - | 1M | - | nervous system |
| [15] | 2×2(φ) | No | ultrasound | - | 1.5M | - | skeleton system |
| [16] | 5(φ) | No | ultrasound | - | 40K | ~1.25mV | skeleton system |
| [17] | 2×1.5(φ) | No | ultrasound & magnetic | - | - | ~4.5V | skeleton system |
| [18] | 11×2(φ) | No | pressure | - | - | - | nervous system |
| [19] | - | No | pressure | - | - | ~300mV | skeleton system |
| [20] | 5×5(φ) | No | pressure | - | - | ~0.36V | skeleton system |
| Challenges | | ※ Require a relatively complex circuit architecture (for device)  ※ Offer a relatively narrow range of tunable output parameters (for material)  ※ Require relatively high-frequency energy input  ※ Provide a limited operational distance | | | | | |
| This work | 3×10×5 | No | ME field | 160 | 60-100 | ~6mV | nervous system |
|  | | - Does not require a complex circuit architecture - Operates at a relatively low input frequency (<100 Hz), compatible with most implantable biomedical applications - Achieves a relatively long working distance (up to 160 mm) without physical contact with the external excitation source - Allows flexible adaptation of the implanted device form factor for application-specific designs, enabling tunable output characteristics | | | | | |

Table S2. PCR primers sequence.

| Gene | Forward Primer (5'–3') | Reverse Primer (5'–3') |
| --- | --- | --- |
| PKC | AAGGACAGTAGTGAGACCTGC | GAGCATTCGGTACAGGAGGAC |
| MAPK | CAGGTGTTCGACGTAGGGC | TCTGGTGCTCAAAAGGACTGA |
| BDNF | TCATACTTCGGTTGCATGAAGG | AGACCTCTCGAACCTGCCC |
| GAP-43 | TGGTGTCAAGCCGGAAGATAA | GCTGGTGCATCACCCTTCT |
| PKA | AGATCGTCCTGACCTTTGAGT | GGCAAAACCGAAGTCTGTCAC |
| CREB | AGCAGCTCATGCAACATCATC | AGTCCTTACAGGAAGACTGAACT |
| β3-Tubulin | TAGACCCCAGCGGCAACTAT | GTTCCAGGTTCCAAGTCCACC |
| MAP2 | GCCAGCCTCGGAACAAACA | GCTCAGCGAATGAGGAAGGA |
| PI3K | CGAGAGTGTCGTCACAGTGTC | TGTTCGCTTCCACAAACACAG |
| ERK | TCAGATGAATTTTCGTTGGCAGA | GAGCACTTGGGTTACTCCACG |
| TRPC1 | TACGGTTGTCAGTCCGCAGA | TCGTTTTGGCCGATGATTAAGTA |
| TRPC2 | CTCAAGGGTATGTTGAAGCAGT | GTTGTTTGGGCTTACCACACT |
| TRPC3 | TCGAGAGGCCACACGACTA | CTGGACAGCGACAAGTATGC |
| CAV1.2 | ATGAAAACACGAGGATGTACGTT | ACTGACGGTAGAGATGGTTGC |
| CAV1.4 | GTGGAGAGAATGAGGACGCAA | TCCGAAGCGGGTTGGTTTG |
| NAV1.2 | TTCATGGCTTCCAATCCCTCC | GGTGTCACGTCAGTCTTCTCT |
| NAV1.3 | CAGACCATGTGCCTTATTGTGT | CCGCGATCTGGAGGTTGTT |
| NAV1.5 | ATGGCAAACTTCCTGTTACCTC | CCACGGGCTTGTTTTTCAGC |
| NAV1.6 | ATGGGGTAGGCTCTCCGAG | CCGACTCTGACTTAAACACCTTC |
| KCNA3 | GGGGCATTGCCATTGTGTC | AGGCGGGATAGTCTTTCTCATC |
| GAPDH | GAAGGTGAAGGTCGGAGTC | GAGATGGTGATGGGATTTC |

**References**

[1] Q. Wang, Y. Zhang, H. Xue, et al., "Lead-free dual-frequency ultrasound implants for wireless, biphasic deep brain stimulation*"*, *Nature Communications* **2024**, *15* (1), <https://doi.org/10.1038/s41467-024-48250-z>.

[2] T. Zhang, H. G. Liang, Z. Wang, et al., "Piezoelectric ultrasound energy-harvesting device for deep brain stimulation and analgesia applications*"*, *Science Advances* **2022**, *8* (15), <https://doi.org/10.1126/sciadv.abk0159>.

[3] L. M. Jiang, G. X. Lu, Y. S. Zeng, et al., "Flexible ultrasound-induced retinal stimulating piezo-arrays for biomimetic visual prostheses*"*, *Nature Communications* **2022**, *13* (1), <https://doi.org/10.1038/s41467-022-31599-4>.

[4] J. Charthad, M. J. Weber, T. C. Chang, et al., "A mm-Sized Implantable Medical Device (IMD) With Ultrasonic Power Transfer and a Hybrid Bi-Directional Data Link*"*, *Ieee Journal of Solid-State Circuits* **2015**, *50* (8), 1741-1753, <https://doi.org/10.1109/Jssc.2015.2427336>.

[5] M. Alam, S. Li, R. U. Ahmed, et al., "Development of a battery-free ultrasonically powered functional electrical stimulator for movement restoration after paralyzing spinal cord injury*"*, *Journal of Neuroengineering and Rehabilitation* **2019**, *16*, <https://doi.org/10.1186/s12984-019-0501-4>.

[6] Y. S. Zeng, C. Gong, G. X. Lu, et al., "A programmable and self-adaptive ultrasonic wireless implant for personalized chronic pain management*"*, *Nature Electronics* **2025**, *8* (5), <https://doi.org/10.1038/s41928-025-01374-6>.

[7] G. T. Hwang, H. Park, J. H. Lee, et al., "Self‐Powered Cardiac Pacemaker Enabled by Flexible Single Crystalline PMN‐PT Piezoelectric Energy Harvester*"*, *Advanced Materials* **2014**, *26* (28), 4880-4887, <https://doi.org/10.1002/adma.201400562>.

[8] J. C. Chen, P. Kan, Z. H. Yu, et al., "A wireless millimetric magnetoelectric implant for the endovascular stimulation of peripheral nerves*"*, *Nature Biomedical Engineering* **2022**, *6* (6), 706-716, <https://doi.org/10.1038/s41551-022-00873-7>.

[9] Z. Yu, J. C. Chen, F. T. Alrashdan, et al., "MagNI: A Magnetoelectrically Powered and Controlled Wireless Neurostimulating Implant*"*, *Ieee Transactions on Biomedical Circuits and Systems* **2020**, *14* (6), 1241-1252, <https://doi.org/10.1109/tbcas.2020.3037862>.

[10] P. Chen, C. Cheng, X. Yang, et al., "Wireless Deep Brain Stimulation by Ultrasound-Responsive Molecular Piezoelectric Nanogenerators*"*, *ACS Nano* **2023**, *17* (24), 25625-25637, <https://doi.org/10.1021/acsnano.3c10227>.

[11] P. Chen, C. Xu, P. Wu, et al., "Wirelessly Powered Electrical-Stimulation Based on Biodegradable 3D Piezoelectric Scaffolds Promotes the Spinal Cord Injury*"*, *ACS Nano* **2022**, *16* (10), 16513-16528, <https://doi.org/10.1021/acsnano.2c05818>.

[12] F. Dai, H. Cheng, H. Qi, et al., "Rochelle salt-based biodegradable piezoelectric devices for nerve regeneration and intestinal motility monitoring*"*, *Nature Communications* **2026**, *17* (1), <https://doi.org/10.1038/s41467-026-68930-2>.

[13] H. Wu, H. Dong, Z. Tang, et al., "Electrical stimulation of piezoelectric BaTiO3 coated Ti6Al4V scaffolds promotes anti-inflammatory polarization of macrophages and bone repair via MAPK/JNK inhibition and OXPHOS activation*"*, *Biomaterials* **2023**, *293*, 121990, <https://doi.org/10.1016/j.biomaterials.2022.121990>.

[14] W. R. Jia, T. L. Wang, F. Chen, et al., "Low-Intensity Pulsed Ultrasound Responsive Scaffold Promotes Intramembranous and Endochondral Ossification via Ultrasonic, Thermal, and Electrical Stimulation*"*, *ACS Nano* **2025**, *19* (4), 4422-4439, <https://doi.org/10.1021/acsnano.4c13357>.

[15] C. X. Liu, B. Yu, Z. W. B. Zhang, et al., "LIPUS activated piezoelectric pPLLA/SrSiO composite scaffold promotes osteochondral regeneration through P2RX1 mediated Ca signaling pathway*"*, *Biomaterials* **2025**, *317*, <https://doi.org/10.1016/j.biomaterials.2025.123084>.

[16] X. Zhang, M. Lian, J. Zhou, et al., "Ultrasound-enhanced and cell-traction-induced piezoelectric scaffolds for repairing bone defects*"*, *J Nanobiotechnology* **2025**, *24* (1), 53, <https://doi.org/10.1186/s12951-025-03913-x>.

[17] X. Liu, C. Y. Xue, J. Guo, et al., "Piezoelectric scaffold with enhanced effect drives the healing of osteochondral defects through electromechanical-immune coupling*"*, *Journal of Nanobiotechnology* **2026**, *24* (1), <https://doi.org/10.1186/s12951-026-04131-9>.

[18] H. Q. Zhang, D. W. Lan, B. Q. Wu, et al., "Electrospun Piezoelectric Scaffold with External Mechanical Stimulation for Promoting Regeneration of Peripheral Nerve Injury*"*, *Biomacromolecules* **2023**, *24* (7), 3268-3282, <https://doi.org/10.1021/acs.biomac.3c00311>.

[19] S. Y. Zhang, L. Huang, W. S. Chen, et al., "Piezoelectric hydrogel with self-powered biomechanical stimulation enhances bone regeneration*"*, *Acta Biomaterialia* **2025**, *195*, 117-133, <https://doi.org/10.1016/j.actbio.2025.02.016>.

[20] J. H. Wu, T. J. Chen, Y. Y. Wang, et al., "Piezoelectric Effect of Antibacterial Biomimetic Hydrogel Promotes Osteochondral Defect Repair*"*, *Biomedicines* **2022**, *10* (5), <https://doi.org/10.3390/biomedicines10051165>.
